# Supplementary material for: The effect of a political crisis on performance of community forests and protected areas in Madagascar
Source: Nat Commun. 2024 Apr 5;15:2963. doi: 10.1038/s41467-024-47318-0 (PMC10997648; doi:10.1038/s41467-024-47318-0)
Supplement: Supplementary file 1 — Supplementary Information [file 41467_2024_47318_MOESM1_ESM.pdf]

Supplementary Materials

The effect of a political crisis on performance of community forests and protected areas in Madagascar

Rachel A. Neugarten[1,2*], Ranaivo A. Rasolofoson[3,4], Christopher B. Barrett[5,6], Ghislain Vieilledent[7], Amanda D. Rodewald[2,1]

[1]Department of Natural Resources and Environment, Cornell University, 226 Mann Drive, Ithaca NY 14853 USA
[2]Cornell Lab of Ornithology, Cornell University, 159 Sapsucker Woods Rd, Ithaca, NY 14850
[3]Duke University Marine Laboratory, Nicholas School of the Environment, Duke University, 135 Duke Marine Lab Rd, Beaufort, NC 28516 USA
[4]School of the Environment, University of Toronto, 33 Willcocks Street, Suite 1016V, Toronto, Ontario M5S 3E8, Canada
[5]Charles H. Dyson School of Applied Economics and Management, Cornell University, Ithaca, NY 14853-7801, USA
[6]Jeb E. Brooks School of Public Policy, , Cornell University, Ithaca, NY 14853-7801, USA
[7]AMAP, Université de Montpellier, CIRAD, CNRS, INRAE, IRD, Montpellier, France

*Corresponding author

Table of Contents

Table of Contents 2

S1. Test of parallel trends in the pre-crisis period 4

S2. Two-period difference-in-differences analysis 4

Supplemental Figures 6

Fig. S1. Community Forest Management areas (CFM), protected areas administered by Madagascar National Parks (MNP), and other System of Protected Areas (SAPM) 6

Fig. S2. Map of forest cover and change in Madagascar 2000-2020 7

Fig. S3a-d Maps of covariates used for matching 10

Fig. S3e-h Maps of covariates used for matching, continued ....**Error! Bookmark not defined.**

e) Elevation (meters) f) slope (percent), g) annual average precipitation 1970-2000 (mm/year), and h) distance to forest edge in the baseline year (2005) (meters) 9

Fig. S3i-k Maps of covariates used for matching, continued ....**Error! Bookmark not defined.**

i) Suitability for rice agriculture (index of suitability) j) vegetation zone (Eastern humid forest, western deciduous forest, or southern deciduous spiny forest), k) population density in the baseline year (2005) (people per square kilometer). 10

Fig. S4. Example of sample points, before matching 11

Fig. S5. Example of sample points, after matching 12

Fig. S6. Match balance results 14

Fig. S7a-d. Maps of time-variant covariates used in event study analysis 16

Fig. S7e-h. Maps of time-variant covariates used in event study analysis (continued)..... **Error! Bookmark not defined.**

Fig. S8. Maps of commune-level index of development (left) and index of level of security / risk of theft (right) used for exploring heterogeneity of effects 17

Fig. S9. Event study model 1: Effect of interaction between CFM and years post crisis on annual deforestation 18

Fig. S10. Effect of distance from urban centers 19

Fig. S11. Results of test of parallel trends in the pre-crisis period 20

Supplemental Tables 21

Table S1. Forest cover in Madagascar 2000-2020 21

Table S2. Event study model 1 (all CFM, 90 m resolution) 21

Table S3. Event study model 1 variation (renewed CFM, 90 m resolution) 23

Table S4. Event study model 1 variation (all CFM, 270 m resolution) 24

Table S5. Event study model 2 (interaction term for distance from urban center) (all CFM, 90 m) 25

Table S6. Event study model 3 (interaction term for level of development, all CFM, 90 m) .. 27

Table S7. Event study model 4 (interaction term for level of security, all CFM, 90m) 29

Table S8. Event study model 5 (interaction term for population density, all CFM, 90m) 30

Table S9. Results of test of parallel trends in the pre-crisis period 32

Table S10. Results of two-period difference-in-differences analysis, all CFM, 90m resolution 33

Supplemental References 34

S1. Test of parallel trends in the pre-crisis period

A key assumption of our analysis is that, in the absence of the political crisis, deforestation trends would have been similar in community forest management (CFM) and protected areas administered by Madagascar National Parks (MNP) (parallel trends assumption). The event study analysis controls for any difference in pre-crisis trends. Nonetheless, we tested this assumption by conducting a statistical test of significance of deforestation trends in CFM and matched MNP forest areas in the pre-crisis period. While this test was not necessary for our analysis, we were curious whether trends in the pre-crisis period were indeed similar in CFM and MNP. For the parallel trends test, we compared deforestation outcomes in CFM and matched MNP areas but focused only on the pre-crisis years (2005-2009) (Equation 3).

$$Y_{it} = \beta_1 CFM_i + \tau_1 year_t + \tau_2 year_t CFM_i + \psi X_{it} + \mu_i + \varepsilon_{it} \qquad (3)$$

Where all variables are defined the same way as in Equation 2, above, except for $year_t$ (t=2005-2009 only). We used all the same time-variant controls described above, as well as individual fixed effects for each forest grid cell, and clustered standard errors at the site level.

We found no significant difference in deforestation trends between CFM and MNP in the pre-crisis years (2005-2009) (Fig. S11, Table S9). Both CFM and MNP were negatively associated with deforestation in the pre-crisis period (that is, they experienced less deforestation over time). This gives us even greater confidence in our findings of relative performance during and after the crisis period. Fig. S11 indicates that deforestation in CFM was declining faster than in MNP in the pre-crisis period, which would create a downward bias in any estimated difference in effect between CFM and MNP during or after the crisis. This indicates that our estimated difference in performance in the post-crisis period are, if anything, conservative.

S2. Two-period difference-in-differences analysis

We also conducted a two-period difference-in-differences (DiD) analysis, comparing the pre-crisis period (2005-2009) to the crisis period (2010-2014). Statistical matching combined with DiD are commonly used in the conservation impact evaluation literature[1–7]. We note that our event study model is a form of a DiD model but has two advantages: it controls for differences in deforestation trends in CFM and MNP in the pre-crisis period, and it allows us to explore annual differences in the effect of the crisis in each year (rather than only a pre-crisis and a crisis effect.) We nonetheless provide the results of the two-period DiD model here.

The equation describing the two-period DiD model is:

$$Y_{ip} = \beta_1 CFM_i + \tau_1 period_p + \tau_2 period_p CFM_i + \psi X_{ip} + \mu_i + \varepsilon_{ip} \qquad (4)$$

Where all the variables are defined the same way as above, except for "period" which takes the value 0 for the pre-crisis period (2005-2009) and 1 for the crisis period (2010-2014). The outcome variable, Y_{ip} now represents deforestation in a given forest grid cell i in a given period p. In other words, deforestation is measured as the total percent of the grid cell that experienced forest cover loss over the four-year pre-crisis period (2005-2009) or in the four-year crisis period

(2010-2014). We included individual fixed effects for each forest grid cell, and clustered standard errors at the site level. The coefficient of interest (τ_2) is the estimated effect of the interaction of CFM and crisis_period.

The results of the two-period DiD model are provided in Table S10. As shown in Table S10, the coefficient of interest is positive but not statistically significant, indicating that the effect of the crisis on CFM performance during the crisis period was not significant, consistent with the results of the event study model.

Supplemental Figures

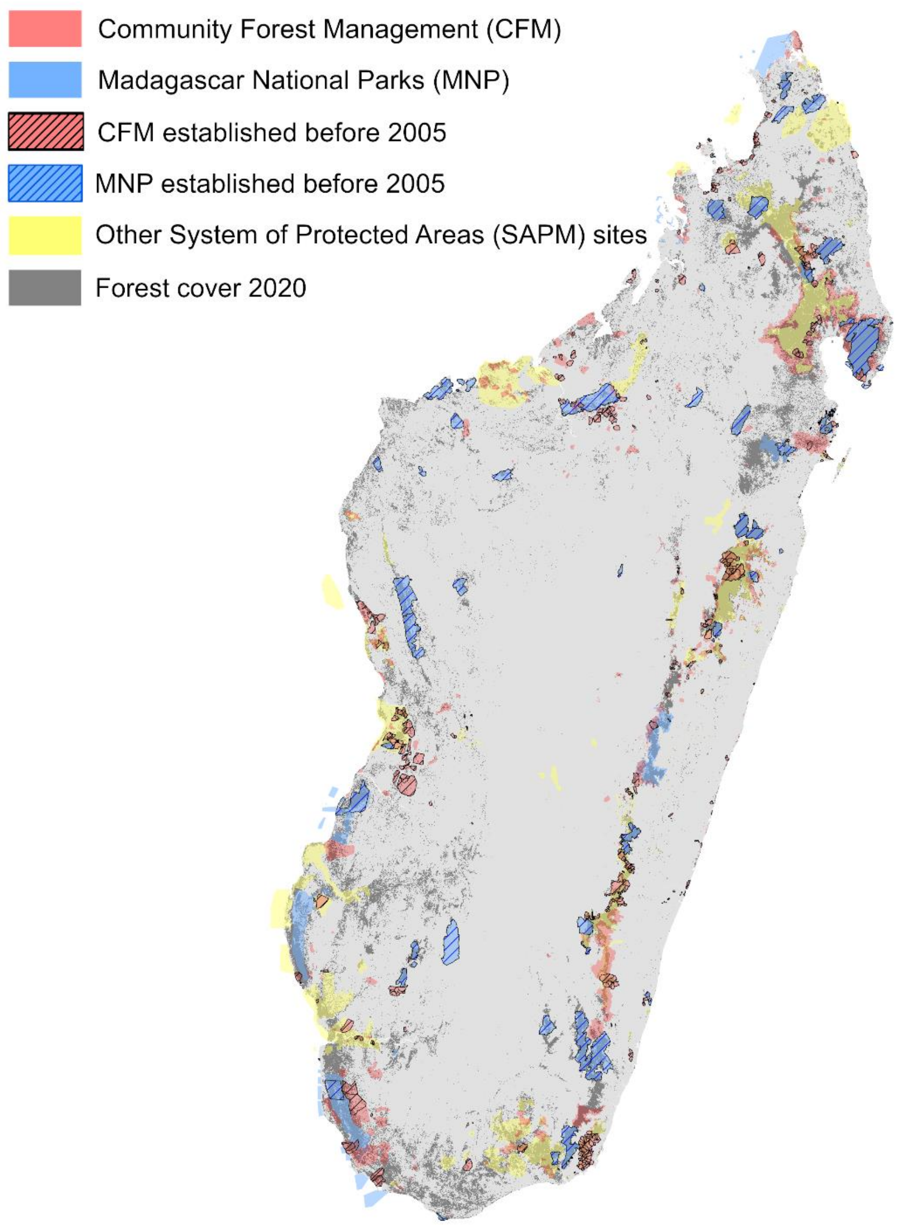

Fig. S1. Community Forest Management areas (CFM), protected areas administered by Madagascar National Parks (MNP), and other System of Protected Areas (SAPM)

Community Forest Management areas (CFM) (red), protected areas administered by Madagascar National Parks (MNP) (blue), and protected areas administered by other agencies (yellow). Only CFM and MNP established before 2005 (red with black hatching, blue with blue hatching) were included in the analysis. Forest cover 2020 (dark gray).

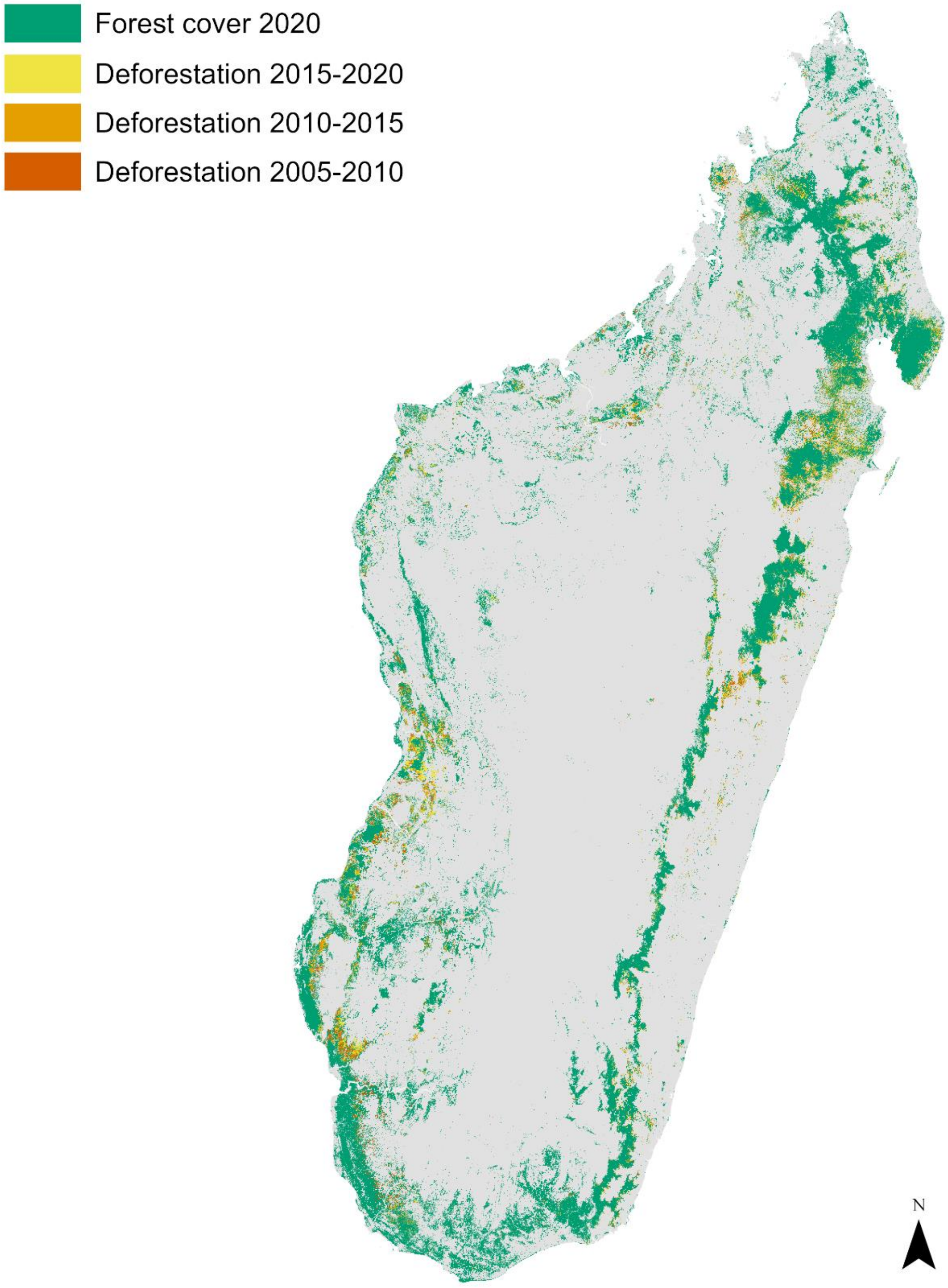

Fig. S2. Map of forest cover and change in Madagascar 2000-2020

Madagascar's forest cover in 2020 (green) and tree cover loss 2005-2010 (red), 2010-2015 (orange), and 2015-2020 (yellow)

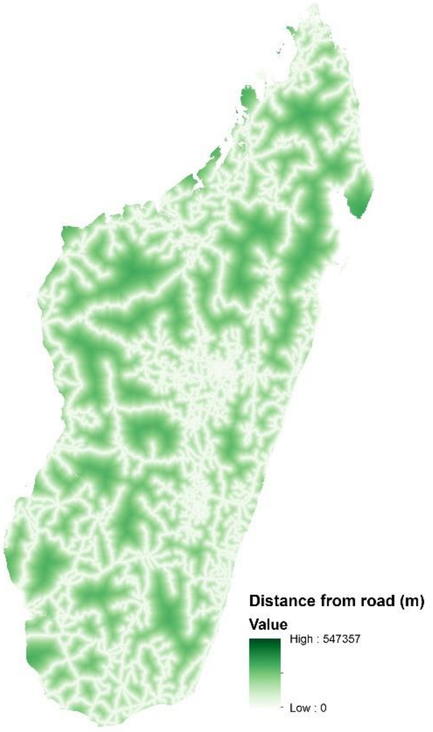
Distance from road (m)
Value
High : 547357
Low : 0

a)

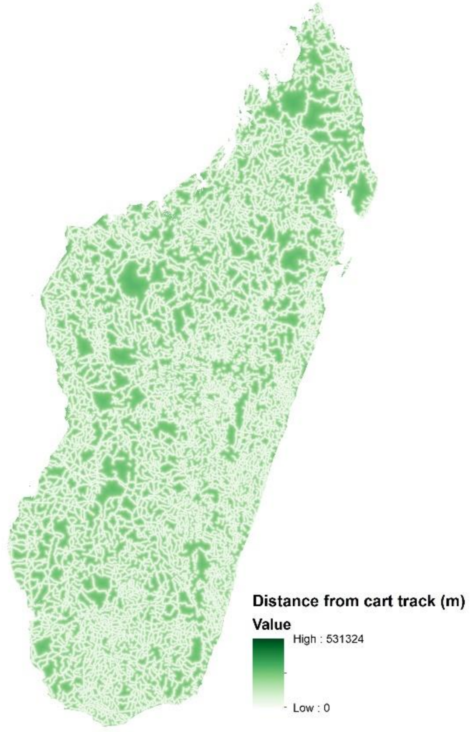
Distance from cart track (m)
Value
High : 531324
Low : 0

b)

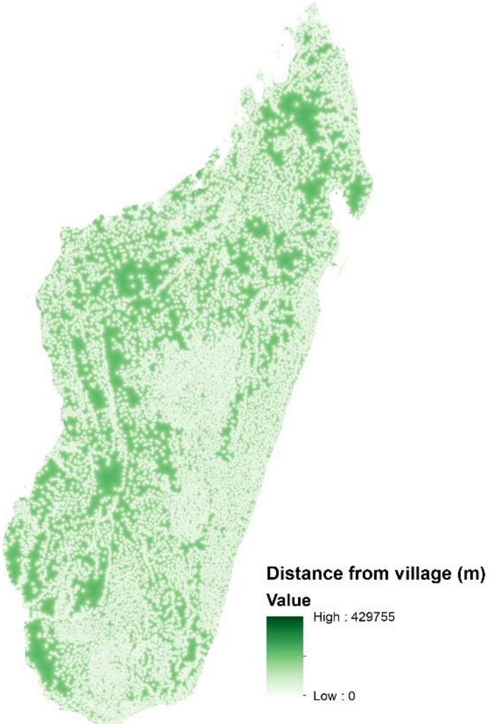
Distance from village (m)
Value
High : 429755
Low : 0

c)

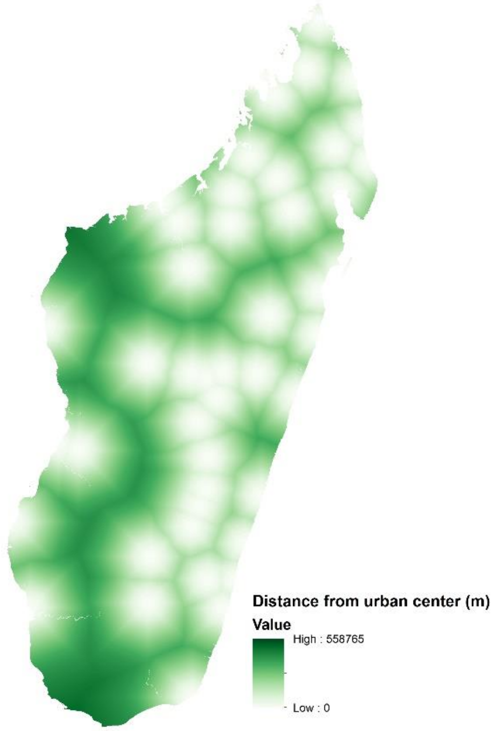
Distance from urban center (m)
Value
High : 558765
Low : 0

d)

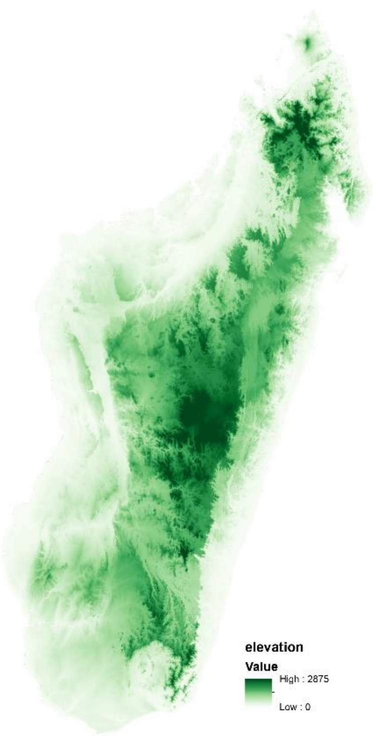
elevation
Value
High : 2875
Low : 0

e)

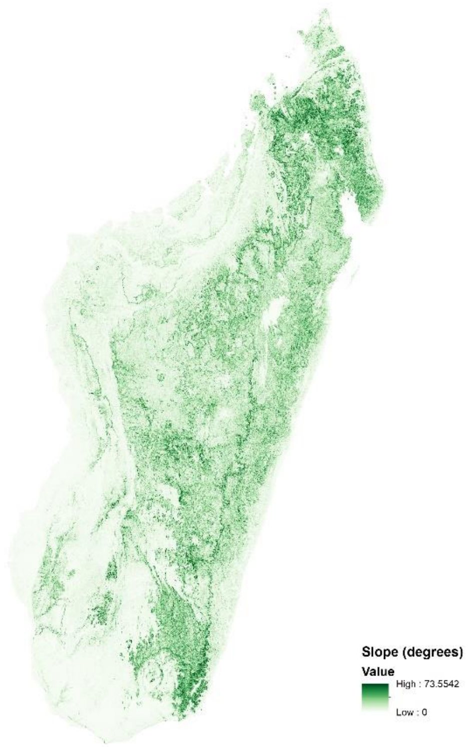
Slope (degrees)
Value
High : 73.5542
Low : 0

f)

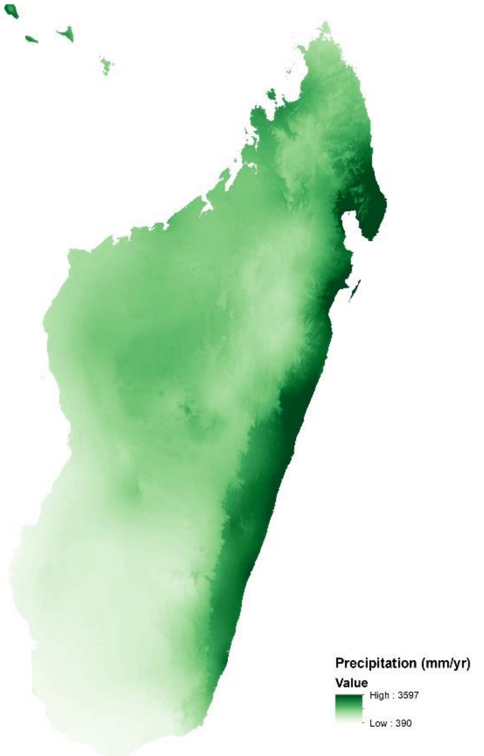
Precipitation (mm/yr)
Value
High : 3597
Low : 390

g)

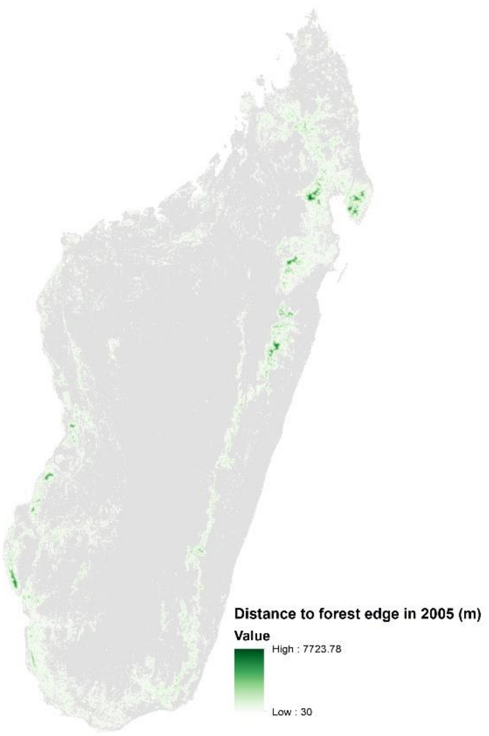
Distance to forest edge in 2005 (m)
Value
High : 7723.78
Low : 30

h)

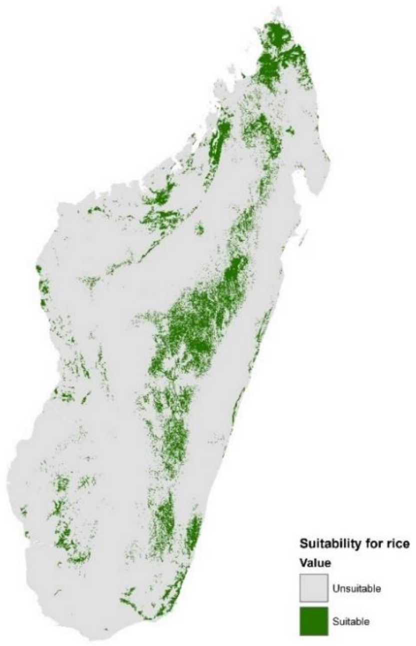

i)

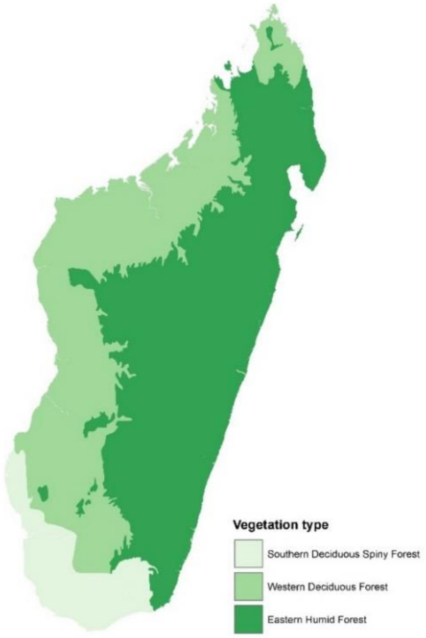

j)

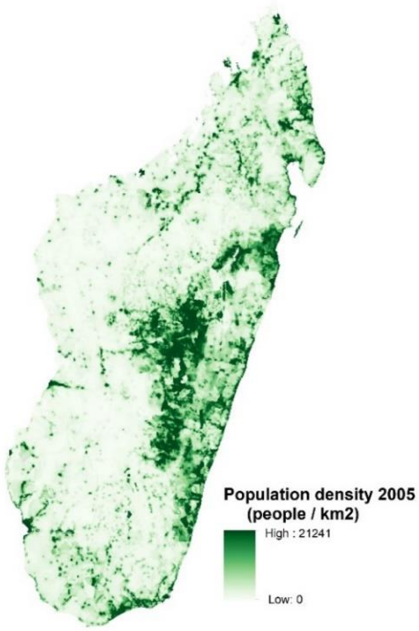

k)

Fig. S3 Maps of covariates used for matching
Distance from a) nearest road, b) nearest cart track, c) nearest village, and d) urban center, all measured in meters; e) elevation (meters) f) slope (percent), g) annual average precipitation 1970-2000 (mm/year), and h) distance to forest edge in the baseline year (2005) (meters), i) Suitability for rice agriculture (index of suitability), j) vegetation zone (Eastern humid forest, western deciduous forest, or southern deciduous spiny forest), k) population density in the baseline year (2005) (people per square kilometer).

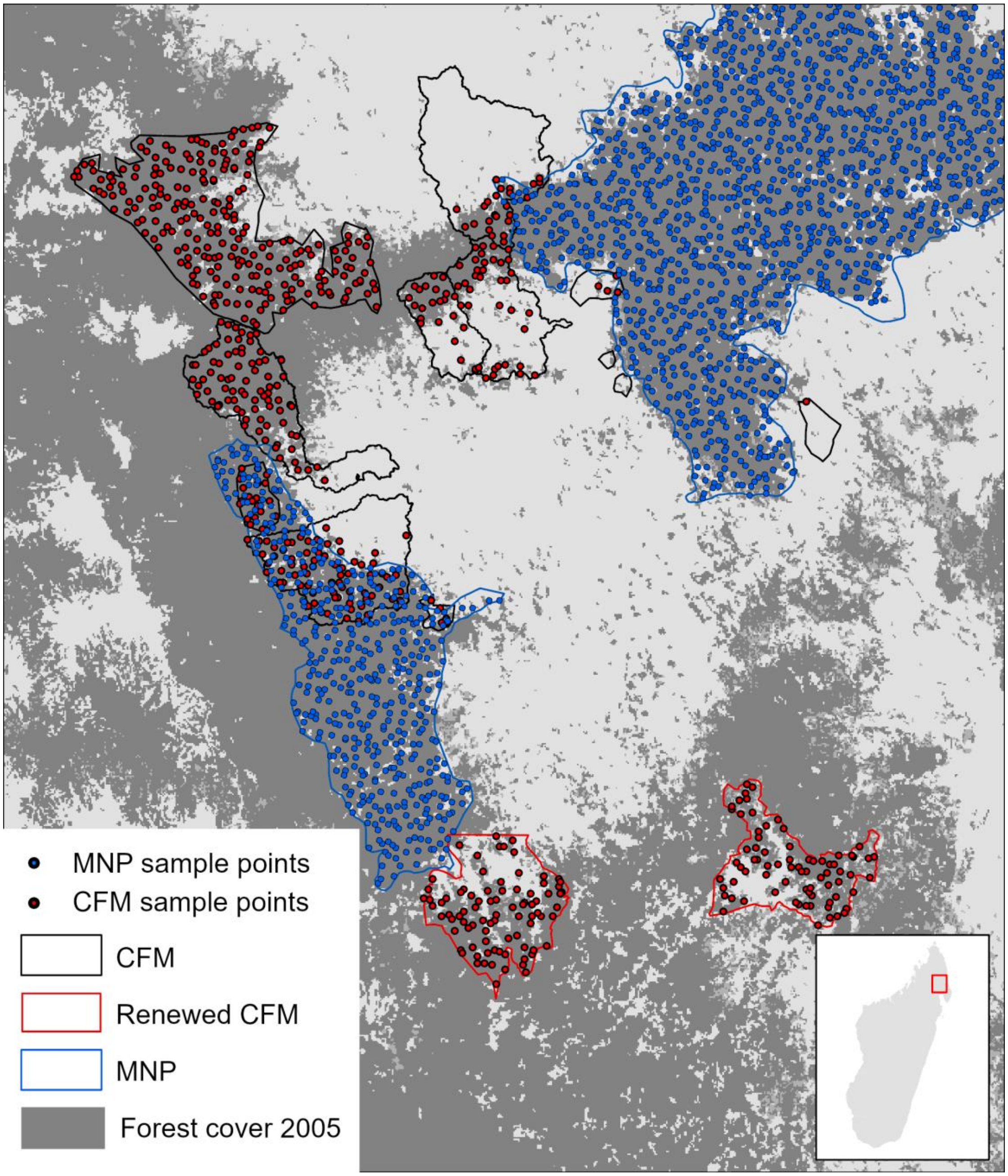

Fig. S4. Example of sample points, before matching

Example of randomly selected (unmatched) sample points within CFM (red) and MNP (blue), baseline (2005) forest cover shown in green. Sample points in overlapping CFM and MNP areas (as shown in center of this map) were excluded from the analysis. Map shows a portion of northeastern Madagascar (Marojejy National Park in the upper right, Anjanaharibe-Sud national park in the center-left.)

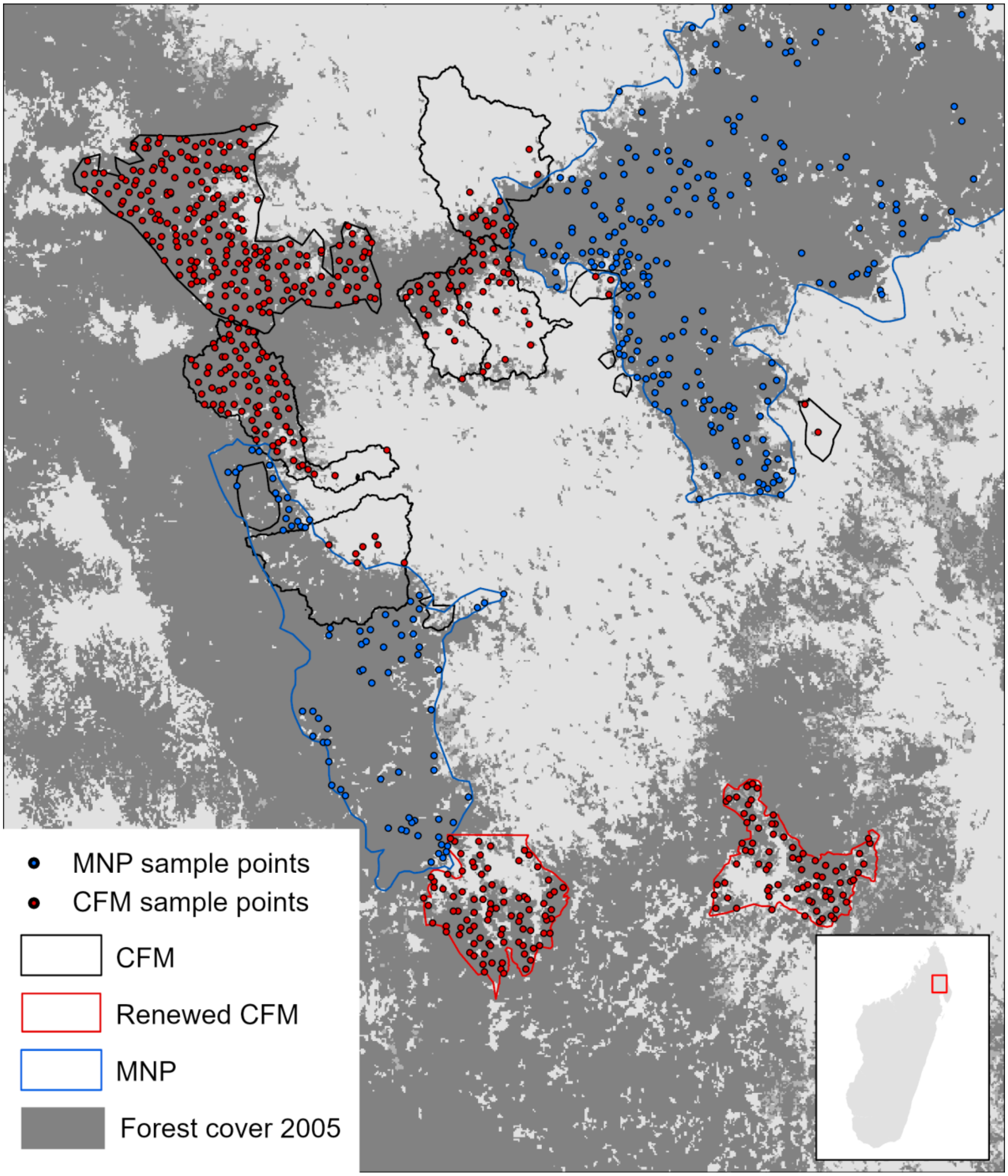

Fig. S5. Example of sample points, after matching

Sample points within CFM (red) and matched points within MNP (blue) with baseline (2005) forest cover shown in green. All CFM sample points were retained, but only MNP sample points that were similar to CFM sample points were included, as these represent a more useful counterfactual.

a)

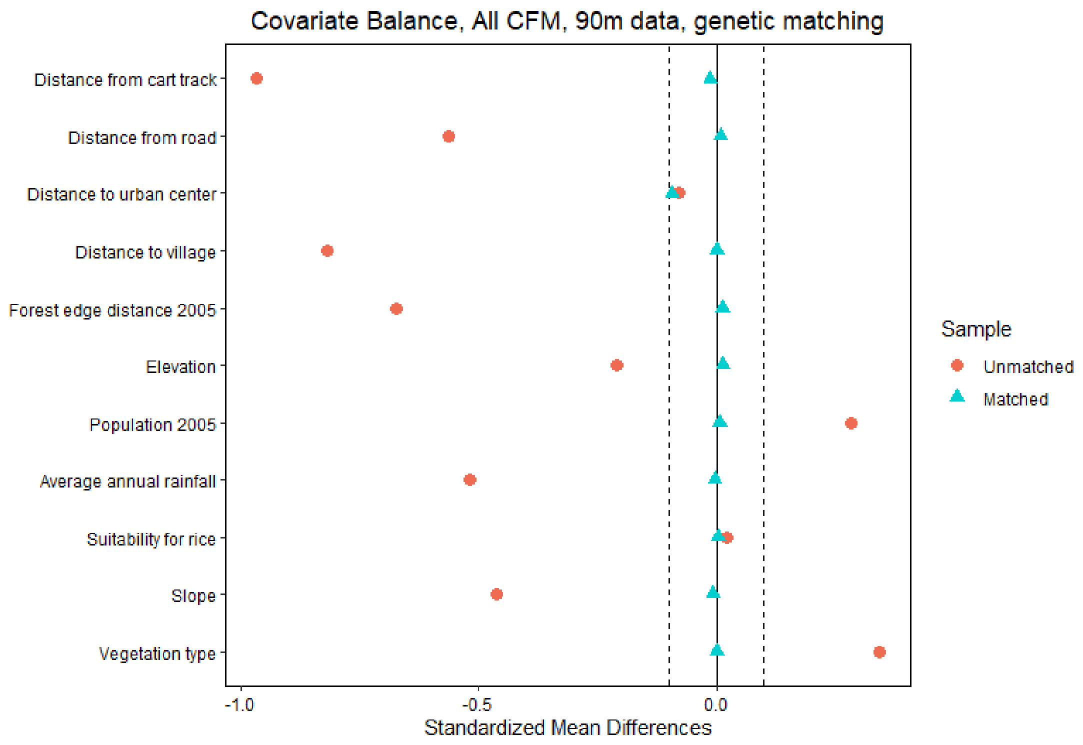
Covariate Balance, All CFM, 90m data, genetic matching
Distance from cart track
Distance from road
Distance to urban center
Distance to village
Forest edge distance 2005
Elevation
Population 2005
Average annual rainfall
Suitability for rice
Slope
Vegetation type
-1.0
-0.5
0.0
Standardized Mean Differences
Sample
Unmatched
Matched

b)

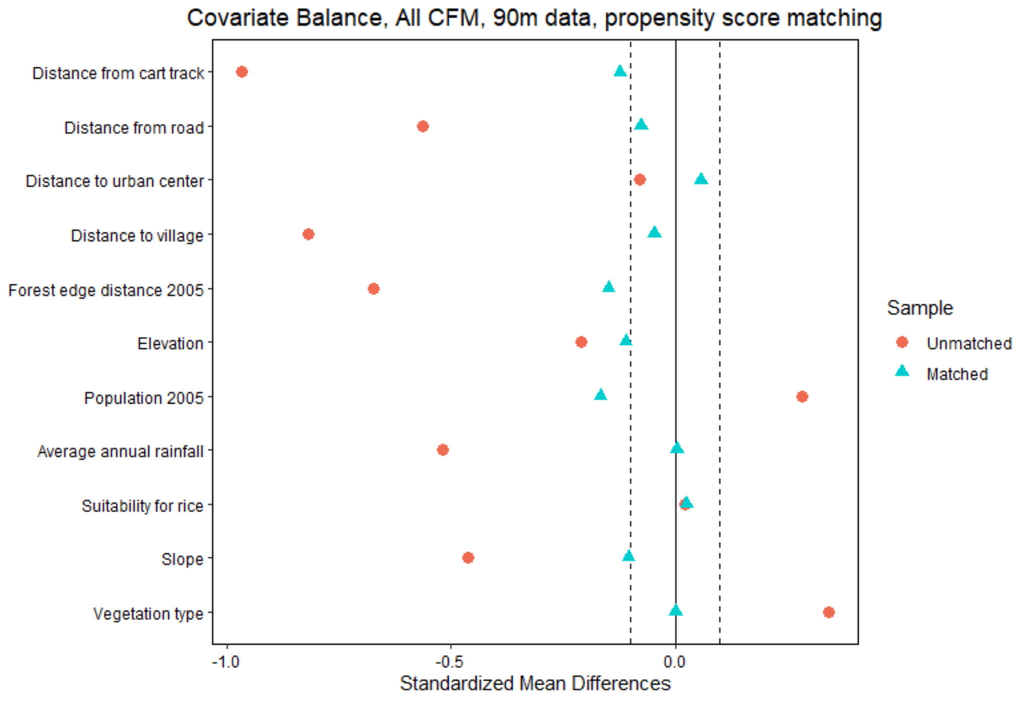
Covariate Balance, All CFM, 90m data, propensity score matching
Distance from cart track
Distance from road
Distance to urban center
Distance to village
Forest edge distance 2005
Elevation
Population 2005
Average annual rainfall
Suitability for rice
Slope
Vegetation type
-1.0
-0.5
0.0
Standardized Mean Differences
Sample
Unmatched
Matched

c)

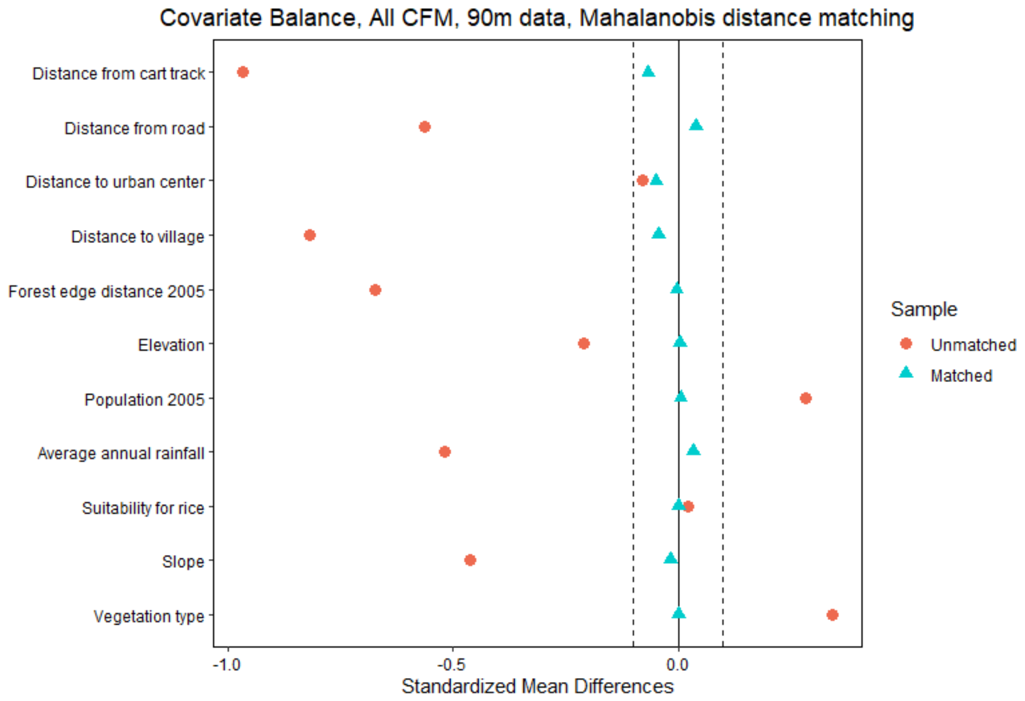

Fig. S6. Match balance results

Standardized mean differences among covariates before matching (red circles) and after matching (blue triangles), using (a) genetic matching, (b) propensity scores, (b) and (c) Mahalanobis distance matching. Black dotted line indicates a standardized mean difference value of 0.1. In all cases, we performed 1:1 matching with replacement, with exact matching on vegetation type, using the “MatchIt” package in R. Results shown are for all CFM data at 90 m resolution; additional matching results (renewed CFM, 270 m) were similar (not shown).

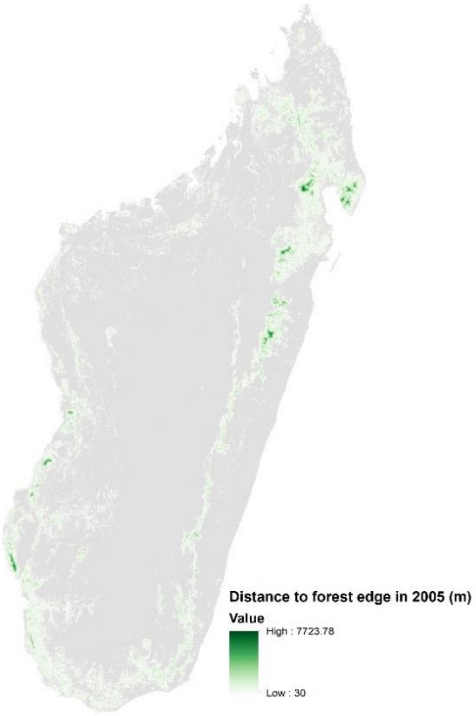
Distance to forest edge in 2005 (m)
Value
High : 7723.78
Low : 30

a)

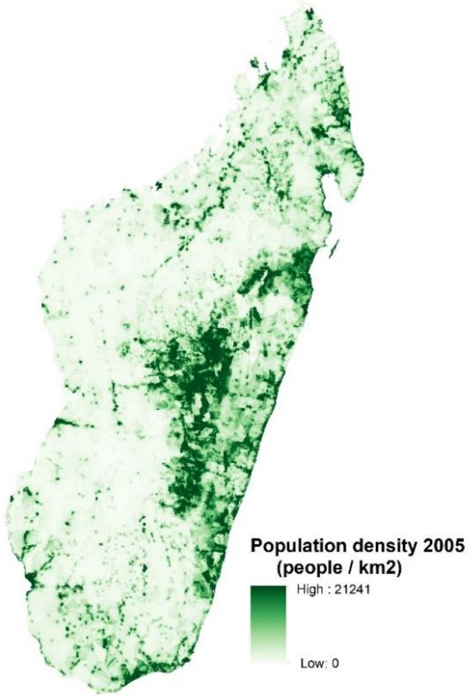
Population density 2005
(people / km2)
High : 21241
Low: 0

b)

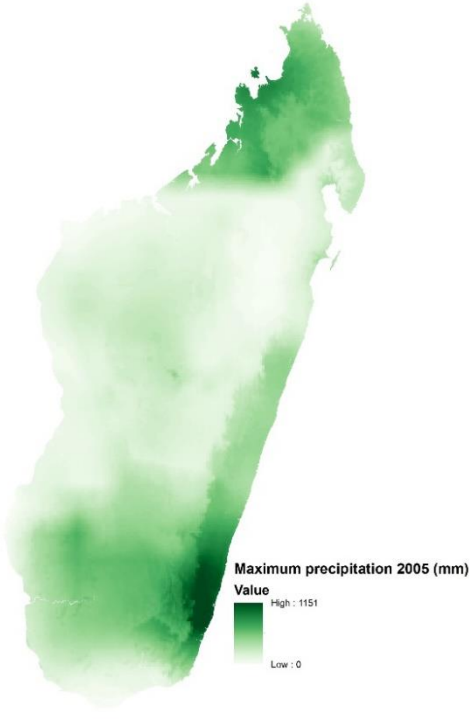
Maximum precipitation 2005 (mm)
Value
High : 1151
Low : 0

c)

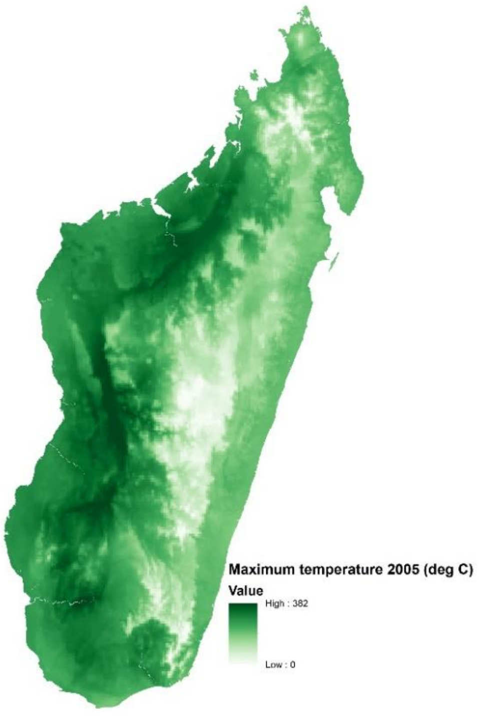
Maximum temperature 2005 (deg C)
Value
High : 382
Low : 0

d)

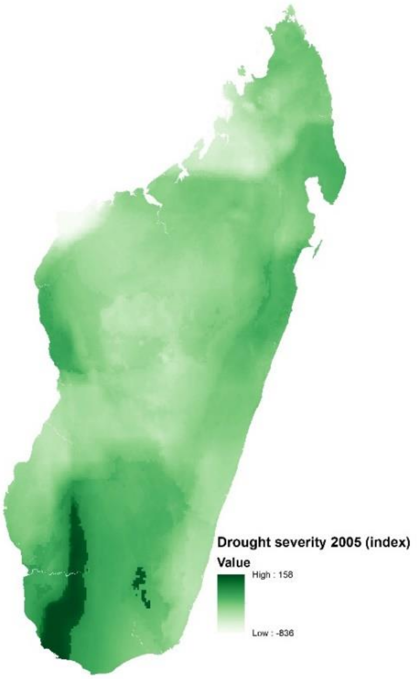

e)

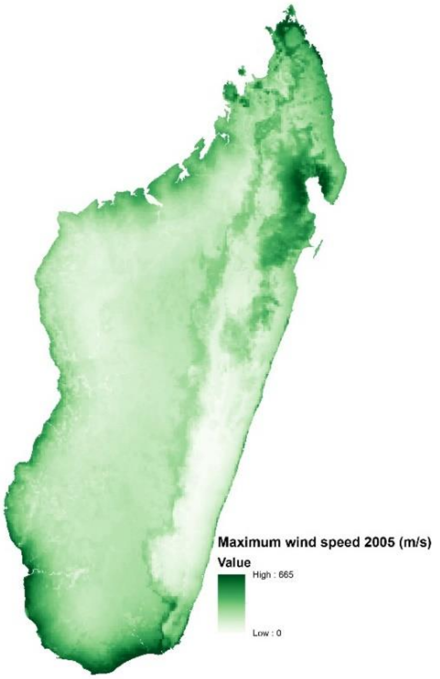

f)

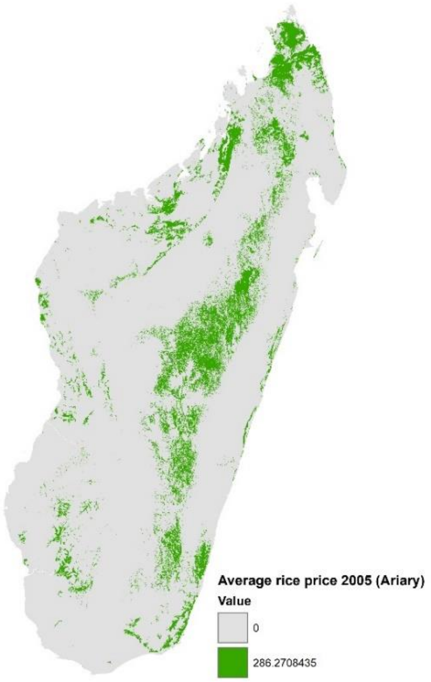

g)

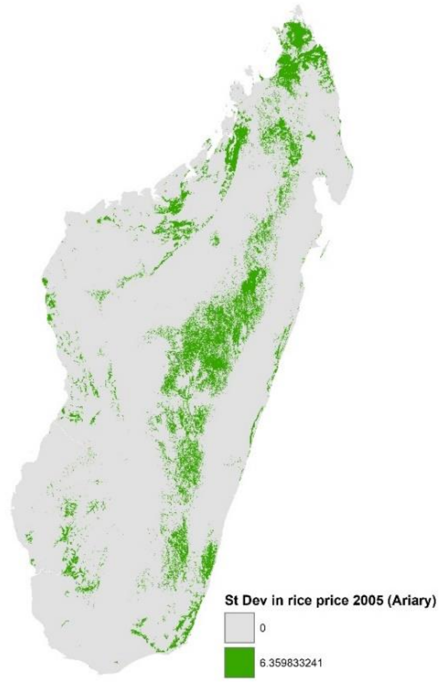

h)

Fig. S7. Maps of time-variant covariates used in event study analysis
a) Distance to forest edge (m), b) population density, c) maximum accumulated precipitation (mm), d) maximum temperature (degrees C), e) Drought severity (Palmer Drought Index), f) maximum wind speed, g) average annual rice price for suitable rice areas (Madagascar currency), h) standard deviation in annual rice price. Maps shown for 2005 only, but full time series includes 2005-2020.

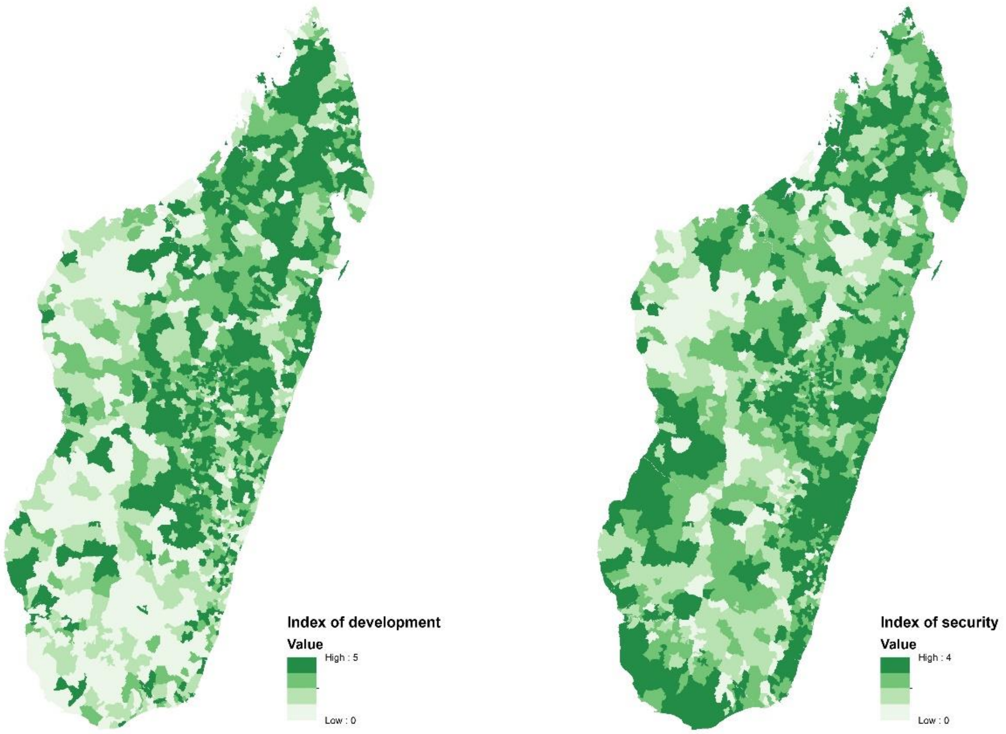

Fig. S8. Maps of commune-level index of development (left) and index of level of security / risk of theft (right) used for exploring heterogeneity of effects

Development level is estimated using an index of material assets. Index of security is based on a single indicator, “Security conditions and risk of theft of property”. Both datasets provided by Wu Yang, Conservation International, and are based on 2007 commune-level data collected by Moser et al. [8].

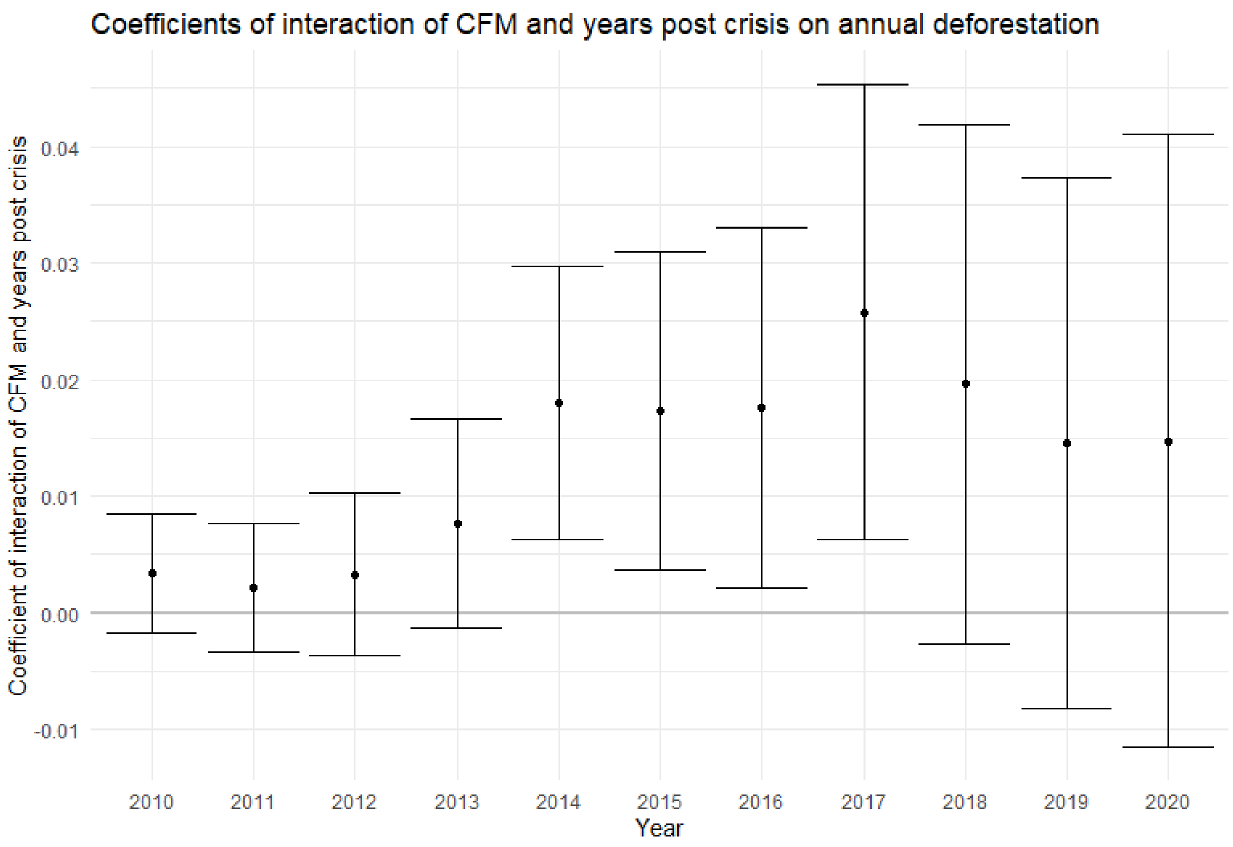

Fig. S9. Event study model 1: Effect of interaction between CFM and years post crisis on annual deforestation

Coefficients of interaction between CFM and years post crisis (2010-2020) on annual deforestation (all CFM, 90 m resolution). Values greater than zero indicate a positive impact on deforestation (worse performance of CFM relative to MNP). Error bars indicate 90% confidence intervals, where standard errors are clustered at the site level. See Equation 2 in the main text (Methods section) for details.

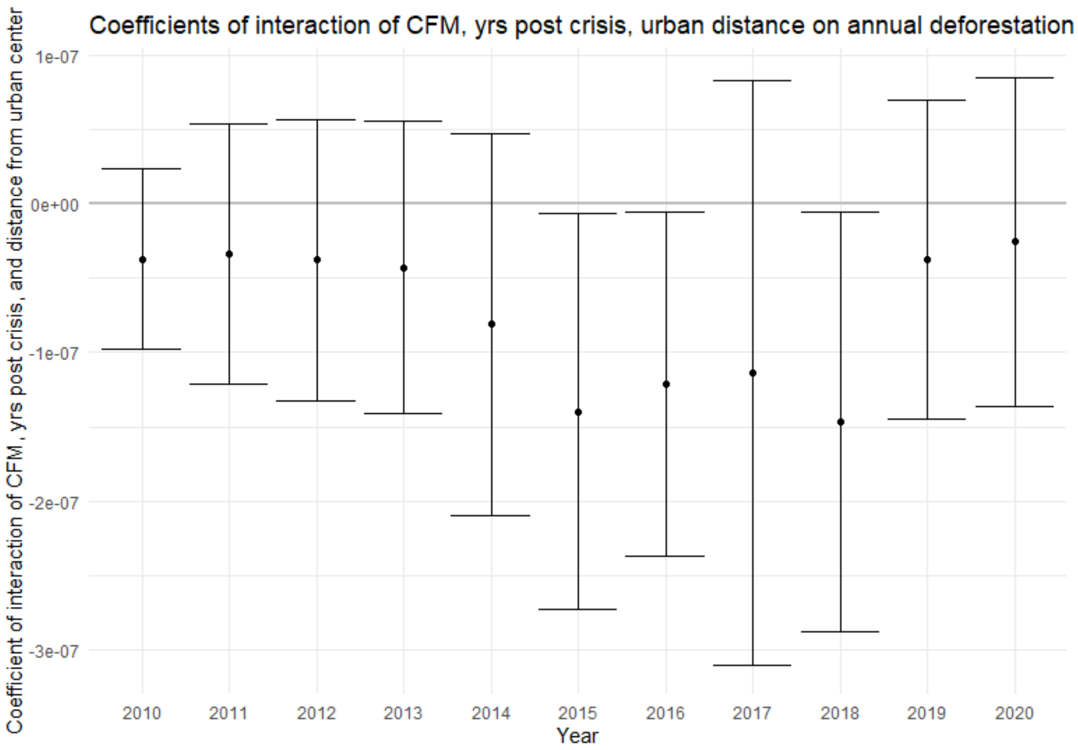

Fig. S10. Effect of distance from urban centers

Effect of interaction of CFM, years post crisis, and distance from urban center. Points below zero indicate a negative association with deforestation. Thus, CFM further from urban centers had lower deforestation than CFM closer to urban centers, and the difference was statistically significant in 2015, 2016, and 2018. See Table S5 for model equation and more details.

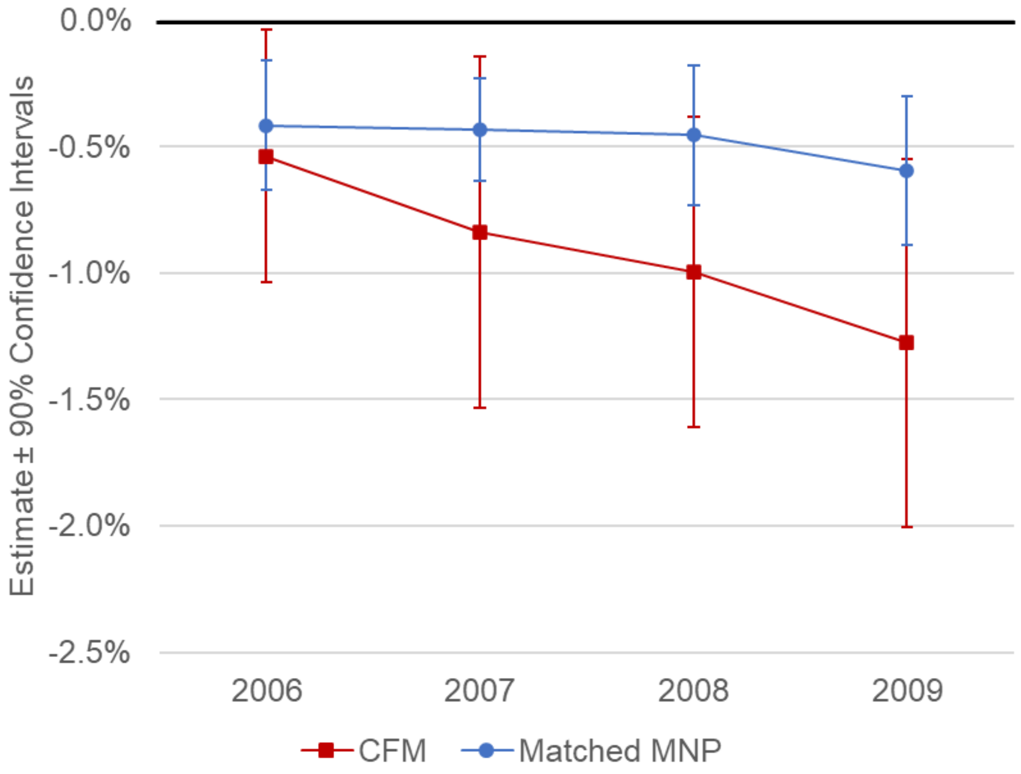

Fig. S11. Results of test of parallel trends in the pre-crisis period

Effects of CFM (red squares) or MNP (blue circles) on annual deforestation, after matching and controlling for time-variant covariates. Estimates below zero indicate a negative association with deforestation (that is, less deforestation over time). Error bars indicate 90% confidence intervals.

Supplemental Tables

Table S1. Forest cover in Madagascar 2000-2020

Forest cover in protected areas administered by Madagascar National Parks and established prior to 2005 (MNP), Community Forest Management areas established before 2005 (CFM), CFM for which contracts were renewed (a sub-set of all CFM sites), other protected and unprotected forests (Other forest) and total national forest cover (Total). “Other forest” includes unprotected forests as well as CFM established after 2005 and protected areas administered by NGOs or agencies other than MNP. “Total” includes all categories. Forest area in each year is reported in hectares.

Year	MNP	CFM	(Renewed CFM)	Other forest	Total
2000	1,213,627	499,526	257,502	8,168,202	9,881,355
2001	1,211,591	498,262	256,892	8,138,936	9,848,789
2002	1,209,917	495,908	255,860	8,098,391	9,804,216
2003	1,209,114	491,744	254,291	8,053,920	9,754,778
2004	1,207,543	490,422	253,577	8,021,703	9,719,669
2005	1,206,329	487,901	251,933	7,974,623	9,668,854
2006	1,205,279	483,110	248,518	7,918,200	9,606,589
2007	1,201,077	478,852	245,987	7,839,204	9,519,133
2008	1,199,491	475,333	244,109	7,779,495	9,454,320
2009	1,197,905	472,293	242,327	7,705,125	9,375,323
2010	1,194,180	468,103	239,833	7,650,085	9,312,368
2011	1,190,754	464,297	237,909	7,570,724	9,225,775
2012	1,187,099	461,221	236,495	7,509,951	9,158,271
2013	1,183,056	455,830	233,692	7,414,854	9,053,740
2014	1,174,892	444,351	228,366	7,248,476	8,867,719
2015	1,169,980	435,157	223,672	7,153,093	8,758,230
2016	1,162,485	426,345	218,622	7,047,973	8,636,803
2017	1,151,026	410,458	210,180	6,874,295	8,435,778
2018	1,140,665	398,568	204,350	6,739,595	8,278,828
2019	1,132,449	391,280	201,721	6,640,802	8,164,530
2020	1,127,322	385,697	199,358	6,558,665	8,071,684

Table S2. Event study model 1 (all CFM, 90 m resolution)

Our first event study model takes the form (Equation 2, also described in the main text):

$$Y_{it} = \beta_1 CFM_i + \tau_1 year_t + \tau_2 year_t CFM_i + \gamma YearsPostCrisis_t + \delta CFM_i YearsPostCrisis_t + \psi X_{it} + \mu_i + \varepsilon_{it} \qquad (2)$$

All variables are defined in the main text (Methods section).

OLS estimation, Dependent variable: Annual deforestation
Observations: 372,032
Fixed-effects: individual sample points: 23,252
Standard-errors: Clustered (site level)

Variable	Estimate	Standard error	Statistic	p-value	Significance
Year	-7.79E-04	2.79E-04	-2.796	0.005	**
2010	2.29E-03	1.19E-03	1.92	0.056	.
2011	5.56E-03	1.77E-03	3.138	0.002	**
2012	3.97E-03	1.29E-03	3.079	0.002	**
2013	4.28E-03	1.65E-03	2.588	0.01	*
2014	7.34E-03	2.33E-03	3.149	0.002	**
2015	6.61E-03	2.30E-03	2.871	0.004	**
2016	7.95E-03	2.68E-03	2.967	0.003	**
2017	1.45E-02	5.12E-03	2.827	0.005	**
2018	1.56E-02	6.28E-03	2.48	0.014	*
2019	1.09E-02	3.49E-03	3.128	0.002	**
2020	1.06E-02	5.27E-03	2.009	0.045	*
Distance from forest edge	-5.32E-05	8.45E-06	-6.296	0	***
Population density	-5.21E-06	4.07E-05	-0.128	0.898	
Average rice price	-5.41E-09	3.57E-09	-1.514	0.131	
Standard deviation in rice price	2.16E-08	1.34E-08	1.615	0.107	
Drought severity (-)	-3.46E-06	1.94E-06	-1.787	0.075	.
Maximum precipitation	5.20E-06	2.90E-06	1.792	0.074	.
Maximum temperature	-6.50E-05	8.04E-05	-0.808	0.42	
Maximum wind speed	-6.46E-06	1.22E-05	-0.53	0.597	
CFM:Year	-1.69E-03	1.15E-03	-1.477	0.141	
CFM:2010	3.13E-03	2.53E-03	1.236	0.217	
CFM:2011	1.22E-03	2.99E-03	0.407	0.684	
CFM:2012	3.18E-03	3.56E-03	0.895	0.372	
CFM:2013	7.28E-03	4.59E-03	1.587	0.113	
CFM:2014	1.79E-02	5.94E-03	3.018	0.003	**
CFM:2015	1.70E-02	6.91E-03	2.468	0.014	*
CFM:2016	1.75E-02	7.84E-03	2.237	0.026	*
CFM:2017	2.43E-02	1.02E-02	2.377	0.018	*
CFM:2018	1.84E-02	1.15E-02	1.6	0.11	
CFM:2019	1.45E-02	1.15E-02	1.256	0.21	
CFM:2020	1.48E-02	1.33E-02	1.119	0.264	

Significance codes: 0 '***' 0.001 '**' 0.01 '*' 0.05 '.' 0.1 ' ' 1
RMSE: 0.065427 Adj. R2: 0.024156 Within R2: 0.017623

Table S3. Event study model 1 variation (renewed CFM, 90 m resolution)

This model is identical to the model described above, but the sample of CFM forest pixels were selected only from a sub-set of CFM for which contracts were renewed. Statistical matching was performed separately and the same way as described above, so the matched dataset in this case reflects MNP forest pixels that are similar to renewed CFM forest pixels. The equation is identical to Equation 2. All below model variations were also performed for this renewed CFM dataset, with very similar results (not shown).

Observations: 380,352
Fixed-effects: individual sample points: 23,772
Standard-errors: Clustered (site level)

Variable	Estimate	Standard error	Statistic	p-value	Significance
Year	-3.26E-04	3.61E-04	-0.904	0.367	
2010	-5.67E-04	1.37E-03	-0.414	0.680	
2011	2.64E-03	2.37E-03	1.114	0.267	
2012	8.03E-04	2.10E-03	0.383	0.703	
2013	1.21E-03	2.49E-03	0.485	0.629	
2014	2.47E-03	2.63E-03	0.941	0.348	
2015	2.43E-03	2.67E-03	0.908	0.365	
2016	3.63E-03	3.82E-03	0.949	0.344	
2017	6.21E-03	4.66E-03	1.333	0.185	
2018	8.53E-03	5.69E-03	1.499	0.136	
2019	5.68E-03	4.90E-03	1.160	0.248	
2020	3.66E-03	5.69E-03	0.643	0.521	
Distance from forest edge	-4.60E-05	1.02E-05	-4.529	0.000	***
Population density	-4.33E-05	8.93E-05	-0.485	0.629	
Average rice price	-4.11E-09	5.03E-09	-0.817	0.415	
Standard deviation in rice price	3.70E-08	2.74E-08	1.353	0.178	
Drought severity (-)	-5.74E-06	3.10E-06	-1.854	0.066	.
Maximum precipitation	6.39E-06	3.93E-06	1.626	0.106	
Maximum temperature	-7.29E-05	7.62E-05	-0.957	0.340	
Maximum wind speed	-1.44E-05	1.42E-05	-1.019	0.310	
CFM:Year	-2.18E-03	1.63E-03	-1.335	0.184	
CFM:2010	5.47E-03	3.55E-03	1.541	0.125	
CFM:2011	3.03E-03	3.97E-03	0.763	0.447	
CFM:2012	4.97E-03	4.81E-03	1.034	0.303	
CFM:2013	1.07E-02	6.31E-03	1.690	0.093	.
CFM:2014	1.97E-02	7.44E-03	2.643	0.009	**
CFM:2015	1.98E-02	8.64E-03	2.293	0.023	*
CFM:2016	2.35E-02	1.04E-02	2.254	0.026	*
CFM:2017	3.07E-02	1.18E-02	2.604	0.010	*
CFM:2018	2.45E-02	1.46E-02	1.680	0.095	.

Variable	Estimate	Standard error	Statistic	p-value	Significance
CFM:2019	1.65E-02	1.64E-02	1.003	0.318	
CFM:2020	1.77E-02	1.83E-02	0.970	0.334	

Significance codes: 0 '***' 0.001 '**' 0.01 '*' 0.05 '.' 0.1 ' ' 1
RMSE: 0.062737 Adj. R2: 0.022307 Within R2: 0.016042

Table S4. Event study model 1 variation (all CFM, 270 m resolution)

This model is identical to Event Study Model 1, above, but all analyses (sampling, statistical matching, and event study analysis) were performed at a coarser spatial resolution (270 m, instead of 90 m). The model equation is identical to Equation 2. All of the below variations on the model were also performed, with very similar results (not shown).

OLS estimation, Dependent variable: Annual deforestation
Observations: 370,272
Fixed-effects: individual sample points: 23,142
Standard-errors: Clustered (site level)

Variable	Estimate	Standard error	Statistic	p-value	Significance
Year	-5.85E-04	1.94E-04	-3.019	0.003	**
2010	2.33E-03	1.31E-03	1.778	0.076	.
2011	2.84E-03	1.19E-03	2.392	0.017	*
2012	2.10E-03	9.69E-04	2.165	0.031	*
2013	3.73E-03	1.60E-03	2.328	0.020	*
2014	6.90E-03	2.32E-03	2.975	0.003	**
2015	4.97E-03	1.57E-03	3.159	0.002	**
2016	5.36E-03	2.29E-03	2.335	0.020	*
2017	1.08E-02	4.74E-03	2.286	0.023	*
2018	8.64E-03	2.80E-03	3.084	0.002	**
2019	9.92E-03	3.44E-03	2.885	0.004	**
2020	6.41E-03	3.01E-03	2.131	0.034	*
Distance from forest edge	-5.34E-05	7.62E-06	-7.001	0.000	***
Population density	1.04E-05	3.62E-05	0.286	0.775	
Average rice price	1.43E-09	5.54E-09	0.258	0.797	
Standard deviation in rice price	2.84E-09	2.13E-08	0.133	0.894	
Drought severity (-)	-2.92E-06	1.76E-06	-1.658	0.098	.
Maximum precipitation	4.99E-06	2.89E-06	1.724	0.086	.
Maximum temperature	-9.89E-05	6.62E-05	-1.495	0.136	
Maximum wind speed	-1.39E-06	1.16E-05	-0.120	0.905	
CFM:Year	-1.91E-03	1.06E-03	-1.805	0.072	.
CFM:2010	2.76E-03	2.30E-03	1.199	0.231	
CFM:2011	3.64E-03	2.40E-03	1.517	0.130	
CFM:2012	4.98E-03	3.17E-03	1.573	0.117	
CFM:2013	8.73E-03	4.21E-03	2.073	0.039	*

Variable	Estimate	Standard error	Statistic	p-value	Significance
CFM:2014	1.89E-02	5.56E-03	3.406	0.001	***
CFM:2015	1.84E-02	6.10E-03	3.015	0.003	**
CFM:2016	1.95E-02	7.23E-03	2.703	0.007	**
CFM:2017	2.68E-02	9.60E-03	2.792	0.006	**
CFM:2018	2.42E-02	9.57E-03	2.524	0.012	*
CFM:2019	1.59E-02	1.08E-02	1.469	0.143	
CFM:2020	1.83E-02	1.18E-02	1.553	0.121	

Significance codes: 0 '***' 0.001 '**' 0.01 '*' 0.05 '.' 0.1 ' ' 1
RMSE: 0.046067 Adj. R2: 0.065911 Within R2: 0.032132

Table S5. Event study model 2 (interaction term for distance from urban center) (all CFM, 90 m)

This variation on the event study model explores heterogeneity of outcomes based on distance from cities, by including an interaction term for distance from urban center:

$$Y_{it} = \beta_1 CFM_i + \tau_1 year_t + \tau_2 year_t CFM_i + \gamma YearsPostCrisis_t + \delta(CFM_i)(YearsPostCrisis_t) + \theta(UrbanDistance)(YearsPostCrisis_t) + \sigma(CFM_i)(UrbanDistance)(YearsPostCrisis_t) + \psi X_{it} + \mu_i + \varepsilon_{it} \quad (4)$$

All variables are defined as above, the new variable, "*UrbanDistance*" indicates the distance, in meters, of each forest grid cell (observation) from the nearest city[3]. Also see Figure S8.

Results (all CFM, 90 m data):
OLS estimation, Dependent variable: Annual deforestation
Observations: 372,032
Fixed-effects: individual sample points: 23,252
Standard-errors: Clustered (site level)

Variable	Estimate	Standard error	Statistic	p-value	Significance
Year	-7.77E-04	2.71E-04	-2.868	0.004	**
2010	1.48E-03	1.11E-03	1.339	0.181	
2011	5.97E-03	2.26E-03	2.644	0.009	**
2012	4.03E-03	1.24E-03	3.245	0.001	**
2013	4.70E-03	1.74E-03	2.698	0.007	**
2014	7.21E-03	2.19E-03	3.294	0.001	**
2015	5.82E-03	2.31E-03	2.517	0.012	*
2016	8.32E-03	2.83E-03	2.939	0.004	**
2017	2.06E-02	7.73E-03	2.668	0.008	**
2018	1.49E-02	6.11E-03	2.445	0.015	*
2019	1.12E-02	3.87E-03	2.902	0.004	**
2020	1.18E-02	6.14E-03	1.920	0.056	.

Variable	Estimate	Standard error	Statistic	p-value	Significance
Distance from forest edge	-5.23E-05	8.29E-06	-6.316	0.000	***
Population density	-6.02E-07	3.91E-05	-0.015	0.988	
Average rice price	-6.32E-09	3.44E-09	-1.835	0.067	.
Standard deviation in rice price	2.35E-08	1.35E-08	1.743	0.082	.
Drought severity (-)	-3.42E-06	1.97E-06	-1.735	0.084	.
Maximum precipitation	4.32E-06	2.89E-06	1.498	0.135	
Maximum temperature	-4.90E-05	9.11E-05	-0.538	0.591	
Maximum wind speed	-2.55E-06	1.25E-05	-0.204	0.838	
CFM:Year	-1.70E-03	1.16E-03	-1.474	0.141	
CFM:2010	5.44E-03	3.79E-03	1.437	0.151	
CFM:2011	3.18E-03	4.91E-03	0.648	0.517	
CFM:2012	5.50E-03	5.78E-03	0.951	0.342	
CFM:2013	9.84E-03	6.87E-03	1.434	0.152	
CFM:2014	2.28E-02	8.68E-03	2.630	0.009	**
CFM:2015	2.55E-02	9.94E-03	2.568	0.011	*
CFM:2016	2.48E-02	1.05E-02	2.363	0.019	*
CFM:2017	3.08E-02	1.49E-02	2.069	0.039	*
CFM:2018	2.73E-02	1.38E-02	1.977	0.049	*
CFM:2019	1.68E-02	1.40E-02	1.203	0.230	
CFM:2020	1.65E-02	1.57E-02	1.049	0.295	
2010:UrbanDistance	1.24E-08	9.86E-09	1.253	0.211	
2011:UrbanDistance	-6.40E-09	1.60E-08	-0.400	0.689	
2012:UrbanDistance	-1.78E-09	8.91E-09	-0.200	0.842	
2013:UrbanDistance	-6.60E-09	8.62E-09	-0.765	0.445	
2014:UrbanDistance	1.61E-09	1.19E-08	0.135	0.893	
2015:UrbanDistance	1.14E-08	9.42E-09	1.213	0.226	
2016:UrbanDistance	-7.50E-09	1.03E-08	-0.726	0.468	
2017:UrbanDistance	-9.54E-08	6.11E-08	-1.560	0.120	
2018:UrbanDistance	7.65E-09	4.23E-08	0.181	0.857	
2019:UrbanDistance	-6.03E-09	1.64E-08	-0.368	0.713	
2020:UrbanDistance	-1.99E-08	2.18E-08	-0.916	0.360	
CFM:2010:UrbanDistance	-3.71E-08	3.08E-08	-1.205	0.229	
CFM:2011:UrbanDistance	-3.35E-08	4.46E-08	-0.752	0.453	
CFM:2012:UrbanDistance	-3.79E-08	4.81E-08	-0.788	0.431	
CFM:2013:UrbanDistance	-4.27E-08	4.99E-08	-0.856	0.393	
CFM:2014:UrbanDistance	-8.10E-08	6.52E-08	-1.243	0.215	
CFM:2015:UrbanDistance	-1.40E-07	6.76E-08	-2.067	0.039	*
CFM:2016:UrbanDistance	-1.21E-07	5.90E-08	-2.052	0.041	*
CFM:2017:UrbanDistance	-1.14E-07	1.00E-07	-1.137	0.256	

Variable	Estimate	Standard error	Statistic	p-value	Significance
CFM:2018:UrbanDistance	-1.47E-07	7.17E-08	-2.047	0.041	*
CFM:2019:UrbanDistance	-3.73E-08	5.47E-08	-0.683	0.495	
CFM:2020:UrbanDistance	-2.56E-08	5.61E-08	-0.456	0.649	

Significance codes: 0 '***' 0.001 '**' 0.01 '*' 0.05 '.' 0.1 ' ' 1
RMSE: 0.065526 Adj. R2: 0.024955 Within R2: 0.0187

Table S6. Event study model 3 (interaction term for level of development, all CFM, 90 m)

This variation on the event study model explores heterogeneity of outcomes based on level of development of the *fonkontany* (smallest administrative unit of Madagascar):

$$\begin{aligned} Y_{it} = \beta_1 CFM_i + \tau_1 year_t + \tau_2 year_t CFM_i + \gamma YearsPostCrisis_t \\ + \delta(CFM_i)(YearsPostCrisis_t) \\ + \lambda(Development)(YearsPostCrisis_t) \\ + \rho(CFM_i)(Development)(YearsPostCrisis_t) + \psi X_{it} + \mu_i + \varepsilon_{it} \end{aligned} \quad (5)$$

All variables are defined as above, the new variable, "*Development*", an index of material assets, data provided by Wu Yang, Conservation International based on 2007 commune-level data originally collected by Moser et al.[8]. Also see Fig. S8.

OLS estimation, Dependent variable: Annual deforestation
Observations: 372,032
Fixed-effects: individual sample points: 23,252
Standard-errors: Clustered (site level)

Variable	Estimate	Standard error	Statistic	p-value	Significance
Year	-7.53E-04	2.76E-04	-2.725	0.007	**
2010	2.99E-03	1.50E-03	1.995	0.047	*
2011	5.19E-03	2.01E-03	2.581	0.010	*
2012	3.17E-03	1.36E-03	2.337	0.020	*
2013	3.70E-03	1.66E-03	2.233	0.026	*
2014	7.16E-03	2.80E-03	2.553	0.011	*
2015	6.42E-03	2.67E-03	2.407	0.017	*
2016	6.87E-03	2.59E-03	2.656	0.008	**
2017	1.28E-02	6.06E-03	2.109	0.036	*
2018	1.89E-02	9.70E-03	1.950	0.052	.
2019	9.41E-03	3.47E-03	2.708	0.007	**
2020	1.05E-02	6.83E-03	1.539	0.125	
Distance from forest edge	-5.34E-05	8.26E-06	-6.472	0.000	***
Population density	-8.81E-06	3.77E-05	-0.234	0.815	
Average rice price	-5.16E-09	3.44E-09	-1.501	0.134	
Standard deviation in rice price	2.11E-08	1.33E-08	1.592	0.112	

Variable	Estimate	Standard error	Statistic	p-value	Significance
Drought severity (-)	-4.11E-06	2.00E-06	-2.053	0.041	*
Maximum precipitation	4.91E-06	2.96E-06	1.661	0.098	.
Maximum temperature	-9.90E-05	9.68E-05	-1.023	0.307	
Maximum wind speed	-7.77E-06	1.25E-05	-0.623	0.534	
CFM:Year	-1.68E-03	1.14E-03	-1.476	0.141	
CFM:2010	3.23E-03	2.67E-03	1.208	0.228	
CFM:2011	-1.15E-03	2.68E-03	-0.428	0.669	
CFM:2012	1.07E-03	2.80E-03	0.383	0.702	
CFM:2013	4.34E-03	3.79E-03	1.144	0.253	
CFM:2014	1.64E-02	5.63E-03	2.915	0.004	**
CFM:2015	1.71E-02	6.39E-03	2.671	0.008	**
CFM:2016	1.46E-02	6.96E-03	2.098	0.037	*
CFM:2017	2.02E-02	1.03E-02	1.964	0.050	.
CFM:2018	1.23E-02	1.30E-02	0.941	0.347	
CFM:2019	1.18E-02	1.08E-02	1.098	0.273	
CFM:2020	1.06E-02	1.32E-02	0.804	0.422	
2010:Development	-1.61E-03	1.14E-03	-1.415	0.158	
2011:Development	6.48E-04	2.07E-03	0.314	0.754	
2012:Development	1.34E-03	1.34E-03	0.997	0.319	
2013:Development	1.33E-03	9.32E-04	1.430	0.154	
2014:Development	2.76E-04	2.14E-03	0.129	0.897	
2015:Development	2.92E-04	1.40E-03	0.208	0.835	
2016:Development	2.21E-03	1.99E-03	1.112	0.267	
2017:Development	3.45E-03	5.35E-03	0.644	0.520	
2018:Development	-8.03E-03	9.05E-03	-0.887	0.376	
2019:Development	3.72E-03	2.67E-03	1.393	0.165	
2020:Development	-5.38E-04	4.63E-03	-0.116	0.907	
CFM:2010:Development	-6.91E-04	3.41E-03	-0.203	0.840	
CFM:2011:Development	6.49E-03	4.18E-03	1.552	0.122	
CFM:2012:Development	5.75E-03	4.02E-03	1.431	0.153	
CFM:2013:Development	8.06E-03	4.37E-03	1.845	0.066	.
CFM:2014:Development	3.84E-03	6.72E-03	0.571	0.569	
CFM:2015:Development	-3.81E-04	6.08E-03	-0.063	0.950	
CFM:2016:Development	8.06E-03	5.56E-03	1.451	0.148	
CFM:2017:Development	1.13E-02	1.08E-02	1.049	0.295	
CFM:2018:Development	1.57E-02	1.05E-02	1.504	0.133	
CFM:2019:Development	7.54E-03	5.42E-03	1.392	0.165	
CFM:2020:Development	1.11E-02	6.80E-03	1.629	0.104	

Significance codes: 0 '***' 0.001 '**' 0.01 '*' 0.05 '.' 0.1 ' ' 1

RMSE: 0.065538 Adj. R2: 0.024606 Within R2: 0.018349

Table S7. Event study model 4 (interaction term for level of security, all CFM, 90m)

This variation on the event study model explores heterogeneity of outcomes based on level of security / risk of theft of the *fonkontany* (smallest administrative unit of Madagascar):

$$\begin{aligned} Y_{it} = \beta_1 CFM_i + \tau_1 year_t + \tau_2 year_t CFM_i + \gamma YearsPostCrisis_t \\ + \delta(CFM_i)(YearsPostCrisis_t) + \pi(Security)(YearsPostCrisis_t) \\ + \eta(CFM_i)(Security)(YearsPostCrisis_t) + \psi X_{it} + \mu_i + \varepsilon_{it} \end{aligned} \quad (6)$$

All variables are defined as above, the new variable, "*Security*", an indicator of security/risk of theft, used here as a proxy for the level of enforcement (e.g. of forest protection rules). Data provided by Wu Yang, Conservation International based on 2007 commune-level data originally collected by Moser et al. [8].

Results:
OLS estimation, Dependent variable: Annual deforestation
Observations: 372,032
Fixed-effects: individual sample points: 23,252, vegtype: 3
Standard-errors: Clustered (site level)

Variable	Estimate	Standard error	Statistic	p-value	Significance
Year	-7.80E-04	2.85E-04	-2.735	0.007	**
2010	1.47E-03	1.09E-03	1.343	0.180	
2011	6.78E-03	2.12E-03	3.193	0.002	**
2012	4.05E-03	1.29E-03	3.147	0.002	**
2013	3.37E-03	1.60E-03	2.107	0.036	*
2014	6.04E-03	2.11E-03	2.862	0.004	**
2015	6.74E-03	2.78E-03	2.421	0.016	*
2016	9.15E-03	3.16E-03	2.892	0.004	**
2017	1.39E-02	6.63E-03	2.096	0.037	*
2018	2.26E-02	1.05E-02	2.149	0.032	*
2019	1.12E-02	3.73E-03	3.011	0.003	**
2020	1.37E-02	7.76E-03	1.768	0.078	.
Distance from forest edge	-5.36E-05	8.37E-06	-6.410	0.000	***
Population density	-1.27E-05	4.31E-05	-0.296	0.768	
Average rice price	-6.05E-09	3.39E-09	-1.787	0.075	.
Standard deviation in rice price	2.23E-08	1.30E-08	1.714	0.087	.
Drought severity (-)	-3.62E-06	1.97E-06	-1.841	0.066	.
Maximum precipitation	6.41E-06	2.87E-06	2.232	0.026	*
Maximum temperature	-1.83E-05	7.11E-05	-0.257	0.797	
Maximum wind speed	-5.93E-06	1.17E-05	-0.507	0.612	
CFM:Year	-1.69E-03	1.16E-03	-1.465	0.144	
CFM:2010	3.24E-03	3.52E-03	0.920	0.358	
CFM:2011	7.86E-04	4.74E-03	0.166	0.868	

Variable	Estimate	Standard error	Statistic	p-value	Significance
CFM:2012	3.05E-03	5.37E-03	0.568	0.570	
CFM:2013	9.01E-03	6.51E-03	1.384	0.167	
CFM:2014	1.91E-02	8.30E-03	2.300	0.022	*
CFM:2015	1.61E-02	9.37E-03	1.716	0.087	.
CFM:2016	1.42E-02	1.01E-02	1.411	0.159	
CFM:2017	1.35E-02	1.27E-02	1.064	0.288	
CFM:2018	8.48E-03	1.57E-02	0.540	0.589	
CFM:2019	1.19E-02	1.34E-02	0.883	0.378	
CFM:2020	1.06E-02	1.61E-02	0.662	0.509	
2010:Development	1.24E-03	1.57E-03	0.789	0.431	
2011:Development	-2.20E-03	1.96E-03	-1.120	0.264	
2012:Development	1.39E-04	1.21E-03	0.115	0.908	
2013:Development	1.21E-03	9.65E-04	1.256	0.210	
2014:Development	2.20E-03	2.12E-03	1.038	0.300	
2015:Development	-4.50E-04	1.31E-03	-0.343	0.732	
2016:Development	-2.23E-03	2.14E-03	-1.040	0.299	
2017:Development	1.14E-03	5.34E-03	0.214	0.831	
2018:Development	-1.35E-02	9.56E-03	-1.412	0.159	
2019:Development	-1.06E-03	2.38E-03	-0.445	0.656	
2020:Development	-5.70E-03	5.48E-03	-1.040	0.299	
CFM:2010:Development	-1.10E-04	4.38E-03	-0.025	0.980	
CFM:2011:Development	5.05E-04	5.29E-03	0.095	0.924	
CFM:2012:Development	3.11E-04	5.38E-03	0.058	0.954	
CFM:2013:Development	-3.44E-03	5.73E-03	-0.600	0.549	
CFM:2014:Development	-2.25E-03	8.16E-03	-0.276	0.783	
CFM:2015:Development	1.80E-03	7.58E-03	0.237	0.813	
CFM:2016:Development	6.54E-03	7.52E-03	0.869	0.385	
CFM:2017:Development	2.21E-02	1.25E-02	1.773	0.077	.
CFM:2018:Development	1.87E-02	1.22E-02	1.533	0.126	
CFM:2019:Development	5.07E-03	6.71E-03	0.755	0.451	
CFM:2020:Development	7.87E-03	8.39E-03	0.938	0.349	

Significance codes: 0 '***' 0.001 '**' 0.01 '*' 0.05 '.' 0.1 ' ' 1
RMSE: 0.06552 Adj. R2: 0.025152 Within R2: 0.018898

Table S8. Event study model 5 (interaction term for population density, all CFM, 90m)

This variation on the event study model explores heterogeneity of outcomes based on population density:

$$Y_{it} = \beta_1 CFM_i + \tau_1 year_t + \tau_2 year_t CFM_i + \gamma YearsPostCrisis_t + \delta(CFM_i)(YearsPostCrisis_t) + \upsilon(Population)(YearsPostCrisis_t) + o(CFM_i)(Population)(YearsPostCrisis_t) + \psi X_{it} + \mu_i + \varepsilon_{it} \quad (7)$$

All variables are defined as above, the new variable, "*Population*", a measure of population density (people per square kilometer) in the baseline year (2005) [9].

OLS estimation, Dependent variable: Annual deforestation
Observations: 372,032
Fixed-effects: individual sample points: 23,252
Standard-errors: Clustered (site level)

Variable	Estimate	Standard error	Statistic	p-value	Significance
Year	-7.86E-04	2.81E-04	-2.80	0.01	**
2010	2.84E-03	1.48E-03	1.92	0.06	.
2011	5.66E-03	1.92E-03	2.94	0.00	**
2012	2.67E-03	1.51E-03	1.77	0.08	.
2013	4.28E-03	1.78E-03	2.41	0.02	*
2014	8.09E-03	2.60E-03	3.11	0.00	**
2015	6.84E-03	2.62E-03	2.61	0.01	**
2016	7.42E-03	2.95E-03	2.52	0.01	*
2017	1.29E-02	5.23E-03	2.47	0.01	*
2018	1.69E-02	7.79E-03	2.16	0.03	*
2019	1.02E-02	3.75E-03	2.71	0.01	**
2020	1.06E-02	6.26E-03	1.69	0.09	.
Population density	3.52E-06	6.48E-05	0.05	0.96	
Distance from forest edge	-5.31E-05	8.46E-06	-6.28	0.00	***
Average rice price	-5.50E-09	3.60E-09	-1.53	0.13	
Standard deviation in rice price	2.15E-08	1.34E-08	1.61	0.11	
Drought severity (-)	-3.26E-06	1.99E-06	-1.63	0.10	
Maximum precipitation	5.36E-06	2.94E-06	1.82	0.07	.
Maximum temperature	-7.05E-05	8.59E-05	-0.82	0.41	
Maximum wind speed	-6.88E-06	1.15E-05	-0.60	0.55	
CFM:Year	-1.69E-03	1.14E-03	-1.48	0.14	
CFM:2010	3.79E-03	2.74E-03	1.38	0.17	
CFM:2011	5.39E-04	2.82E-03	0.19	0.85	
CFM:2012	5.04E-03	3.22E-03	1.57	0.12	
CFM:2013	7.58E-03	4.20E-03	1.81	0.07	.
CFM:2014	1.96E-02	5.72E-03	3.42	0.00	***
CFM:2015	1.86E-02	6.57E-03	2.83	0.00	**
CFM:2016	1.85E-02	7.35E-03	2.52	0.01	*
CFM:2017	3.03E-02	1.01E-02	2.99	0.00	**

Variable	Estimate	Standard error	Statistic	p-value	Significance
CFM:2018	1.81E-02	1.20E-02	1.51	0.13	
CFM:2019	1.52E-02	1.11E-02	1.37	0.17	
CFM:2020	1.29E-02	1.33E-02	0.97	0.33	
CFM:Population	-1.10E-05	1.46E-04	-0.08	0.94	
2010:Population	-2.40E-05	2.00E-05	-1.20	0.23	
2011:Population	-3.90E-06	2.63E-05	-0.15	0.88	
2012:Population	5.90E-05	3.74E-05	1.58	0.12	
2013:Population	1.26E-06	2.18E-05	0.06	0.95	
2014:Population	-2.97E-05	2.99E-05	-0.99	0.32	
2015:Population	-7.84E-06	2.92E-05	-0.27	0.79	
2016:Population	2.26E-05	4.04E-05	0.56	0.58	
2017:Population	6.00E-05	6.69E-05	0.90	0.37	
2018:Population	-4.48E-05	7.21E-05	-0.62	0.54	
2019:Population	2.62E-05	4.49E-05	0.58	0.56	
2020:Population	1.15E-06	5.46E-05	0.02	0.98	
CFM:2010:Population	-3.09E-05	4.47E-05	-0.69	0.49	
CFM:2011:Population	3.16E-05	5.43E-05	0.58	0.56	
CFM:2012:Population	-8.19E-05	5.90E-05	-1.39	0.17	
CFM:2013:Population	-1.24E-05	6.15E-05	-0.20	0.84	
CFM:2014:Population	-6.67E-05	7.70E-05	-0.87	0.39	
CFM:2015:Population	-6.15E-05	6.85E-05	-0.90	0.37	
CFM:2016:Population	-3.79E-05	7.18E-05	-0.53	0.60	
CFM:2017:Population	-2.26E-04	1.16E-04	-1.95	0.05	.
CFM:2018:Population	1.37E-05	1.01E-04	0.14	0.89	
CFM:2019:Population	-2.40E-05	7.50E-05	-0.32	0.75	
CFM:2020:Population	6.41E-05	9.02E-05	0.71	0.48	

Significance codes: 0 '***' 0.001 '**' 0.01 '*' 0.05 '.' 0.1 ' ' 1
RMSE: 0.065548 Adj. R2: 0.024313 Within R2: 0.018057

Table S9. Results of test of parallel trends in the pre-crisis period

The coefficient of interest is the interaction between CFM and year. Results shown are from all CFM established before 2005, at 90 m spatial resolution.

OLS estimation, dependent variable: annual deforestation
Observations: 116,260
Fixed-effects: individual sample points: 23,252
Standard-errors: Clustered

Variable	Estimate	Standard error	Statistic	p-value	Significance
year2006	-4.17E-03	1.56E-03	-2.674	0.008	**
year2007	-4.35E-03	1.23E-03	-3.530	0.000	***

year2008	-4.55E-03	1.68E-03	-2.702	0.007	**
year2009	-5.95E-03	1.80E-03	-3.309	0.001	**
Distance to forest edge	-1.02E-04	3.14E-05	-3.254	0.001	**
Population density	1.00E-04	8.19E-05	1.223	0.222	
Average rice price	-7.67E-10	5.92E-09	-0.130	0.897	
Standard deviation in rice price	8.91E-09	1.43E-08	0.623	0.534	
Drought severity (-)	2.99E-06	2.95E-06	1.015	0.311	
Maximum precipitation	3.03E-06	4.09E-06	0.742	0.459	
Maximum temperature	-3.07E-05	1.73E-04	-0.178	0.859	
Maximum wind speed	-3.83E-05	2.48E-05	-1.546	0.123	
CFM:year2006	-1.21E-03	3.19E-03	-0.378	0.705	
CFM:year2007	-4.03E-03	4.47E-03	-0.901	0.368	
CFM:year2008	-5.41E-03	4.08E-03	-1.327	0.185	
CFM:year2009	-6.82E-03	4.18E-03	-1.633	0.103	

Significance codes: 0 '***' 0.001 '**' 0.01 '*' 0.05 '.' 0.1 ' ' 1
RMSE: 0.047993 Adj. R2: 0.026602 Within R2: 0.010348

Table S10. Results of two-period difference-in-differences analysis, all CFM, 90m resolution

Variable	Estimate	Standard error	Statistic	p-value	Significance
Crisis_period	1.41E-02	6.11E-03	2.302	0.022	*
Distance from forest edge	-6.33E-05	5.09E-05	-1.244	0.214	
Population density	-4.70E-04	3.76E-04	-1.251	0.212	
Average rice price	-9.43E-08	3.42E-08	-2.756	0.006	**
Drought severity (-)	-7.53E-05	3.93E-05	-1.915	0.056	.
Maximum precipitation	-7.43E-06	5.97E-05	-0.124	0.901	
Maximum temperature	-1.57E-03	1.65E-03	-0.949	0.343	
Maximum wind speed	-1.24E-04	2.12E-04	-0.583	0.560	
CFM:crisis_period	2.17E-03	1.11E-02	0.195	0.845	

Significance codes: 0 '***' 0.001 '**' 0.01 '*' 0.05 '.' 0.1 ' ' 1
RMSE: 0.087646 Adj. R2: 0.016397 Within R2: 0.009474

Supplemental References

1. Blackman, A. & Villalobos, L. Use Forests or Lose Them? Regulated Timber Extraction and Tree Cover Loss in Mexico. *Journal of the Association of Environmental and Resource Economists* **8**, 125–163 (2021).

2. Chervier, C. & Costedoat, S. Heterogeneous Impact of a Collective Payment for Environmental Services Scheme on Reducing Deforestation in Cambodia. *World Development* **98**, 148–159 (2017).

3. Rasolofoson, R. A., Ferraro, P. J., Jenkins, C. N. & Jones, J. P. G. Effectiveness of Community Forest Management at reducing deforestation in Madagascar. *Biological Conservation* **184**, 271–277 (2015).

4. Bauch, S. C., Sills, E. O. & Pattanayak, S. K. Have We Managed to Integrate Conservation and Development? ICDP Impacts in the Brazilian Amazon. *World Development* **64**, S135–S148 (2014).

5. Solis, D., Cronkleton, P., Sills, E. O., Rodriguez-Ward, D. & Duchelle, A. E. Evaluating the Impact of REDD+ Interventions on Household Forest Revenue in Peru. *Frontiers in Forests and Global Change* **4**, (2021).

6. Jones, K. W. *et al.* Measuring the net benefits of payments for hydrological services programs in Mexico. *Ecological Economics* **175**, 106666 (2020).

7. Sharma, B. P. *et al.* Making incremental progress: impacts of a REDD+ pilot initiative in Nepal. *Environ. Res. Lett.* **15**, 105004 (2020).

8. Moser, C., Ralison, E., Randrianjatovo, J. F. & Ravelomanana, S. *Enquête Sur Le Suivi Du Recensement Des Communes de Madagascar Année 2007 (2007 Monitoring Census Survey of Madagascar Communes). Final Report.* (2008).

9. WorldPop. *Global High Resolution Population Denominators Project*. https://dx.doi.org/10.5258/SOTON/WP00674 (2018).
