# Supplementary material for: The effect of a political crisis on performance of community forests and protected areas in Madagascar
Source: Nat Commun. 2024 Apr 5;15:2963. doi: 10.1038/s41467-024-47318-0 (PMC10997648; doi:10.1038/s41467-024-47318-0)
Supplement: Supplementary file 3 — Reporting Summary [file 41467_2024_47318_MOESM3_ESM.pdf]

nature portfolio

Corresponding author(s): Rachel A Neugarten

Last updated by author(s): Mar 16, 2024

Reporting Summary

Nature Portfolio wishes to improve the reproducibility of the work that we publish. This form provides structure for consistency and transparency in reporting. For further information on Nature Portfolio policies, see our Editorial Policies and the Editorial Policy Checklist.

Statistics

For all statistical analyses, confirm that the following items are present in the figure legend, table legend, main text, or Methods section.

n/a	Confirmed	
☐	☒	The exact sample size (*n*) for each experimental group/condition, given as a discrete number and unit of measurement
☐	☒	A statement on whether measurements were taken from distinct samples or whether the same sample was measured repeatedly
☐	☒	The statistical test(s) used AND whether they are one- or two-sided<br>*Only common tests should be described solely by name; describe more complex techniques in the Methods section.*
☐	☒	A description of all covariates tested
☐	☒	A description of any assumptions or corrections, such as tests of normality and adjustment for multiple comparisons
☐	☒	A full description of the statistical parameters including central tendency (e.g. means) or other basic estimates (e.g. regression coefficient) AND variation (e.g. standard deviation) or associated estimates of uncertainty (e.g. confidence intervals)
☐	☒	For null hypothesis testing, the test statistic (e.g. *F*, *t*, *r*) with confidence intervals, effect sizes, degrees of freedom and *P* value noted<br>*Give P values as exact values whenever suitable.*
☒	☐	For Bayesian analysis, information on the choice of priors and Markov chain Monte Carlo settings
☒	☐	For hierarchical and complex designs, identification of the appropriate level for tests and full reporting of outcomes
☐	☒	Estimates of effect sizes (e.g. Cohen's *d*, Pearson's *r*), indicating how they were calculated

Our web collection on statistics for biologists contains articles on many of the points above.

Software and code

Policy information about availability of computer code

Data collection	No software was used for data collection.
Data analysis	Data was analyzed using ArcGIS Desktop v10.7 (Esri 2019), QGIS Desktop 3.26.3 (open source: https://qgis.org/en/site/), and R statistical software (v4.2.2) (open source: https://cran.r-project.org/). R code used in this analysis has been made available via https://github.com/raenb0/madagascar

For manuscripts utilizing custom algorithms or software that are central to the research but not yet described in published literature, software must be made available to editors and reviewers. We strongly encourage code deposition in a community repository (e.g. GitHub). See the Nature Portfolio guidelines for submitting code & software for further information.

Data

Policy information about availability of data

All manuscripts must include a data availability statement. This statement should provide the following information, where applicable:

- Accession codes, unique identifiers, or web links for publicly available datasets
- A description of any restrictions on data availability
- For clinical datasets or third party data, please ensure that the statement adheres to our policy

The forest cover, deforestation, time-invariant and time-variant covariates data generated in this study have been deposited in the Zenodo database under accession code 10.5281/zenodo.8132923 [https://doi.org/10.5281/zenodo.8132923]. Shapefile polygons for protected areas in Madagascar are available from the

World Database of Protected Areas: https://www.protectedplanet.net/country/MDG The Community Forest Management areas polygon data are available under restricted access as this was what was agreed with the communities when the data was collected, access can be obtained upon reasonable request to Ranaivo Rasolofoson [ranaivo (dot) rasolofoson (at) duke (dot) edu].

Research involving human participants, their data, or biological material

Policy information about studies with human participants or human data. See also policy information about sex, gender (identity/presentation), and sexual orientation and race, ethnicity and racism.

Reporting on sex and gender	NA
Reporting on race, ethnicity, or other socially relevant groupings	NA
Population characteristics	NA
Recruitment	NA
Ethics oversight	NA

Note that full information on the approval of the study protocol must also be provided in the manuscript.

Field-specific reporting

Please select the one below that is the best fit for your research. If you are not sure, read the appropriate sections before making your selection.

☐ Life sciences ☐ Behavioural & social sciences ☒ Ecological, evolutionary & environmental sciences

For a reference copy of the document with all sections, see nature.com/documents/nr-reporting-summary-flat.pdf

Ecological, evolutionary & environmental sciences study design

All studies must disclose on these points even when the disclosure is negative.

Study description	We evaluated the performance of two different conservation interventions, Community Forest Management (CFM) ("treatment") relative to protected areas administered by Madagascar National Parks (MNP) ("control") in terms of their ability to reduce deforestation (outcome) during a 2009-2014 political crisis. We used secondary data on deforestation derived from remote sensing combined with spatial data on a large number of biophysical and socioeconomic covariates (slope, elevation, distance from roads, commodity prices, human population density). We combined statistical matching within an event study analysis to try to isolate the causal effect of CFM relative to MNP on deforestation outcomes 2005-2020.
Research sample	Our sample consisted of 12,000 randomly forest grid cells within CFM, and a matched sample of 12,000 forest grid cells within MNP. The analysis was repeated at two spatial resolutions (90 meters and 270 meters). The population the sample is meant to represent is all forests within Community Forest Management areas (CFM) in Madagascar.
Sampling strategy	Sample and comparison points were generated using the "create spatially balanced points" tool in ArcMap which generates a random sample of points that roughly represent the same proportion of the total study area.
Data collection	All data included here are secondary datasets and were gathered by the lead author from open access sources (e.g. protected areas boundaries from https://www.protectedplanet.net/country/MDG , deforestation data from Google Earth Engine https://developers.google.com/earth-engine/datasets/catalog/UMD_hansen_global_forest_change_2022_v1_10 , climate data also from GEE: https://developers.google.com/earth-engine/datasets/catalog/IDAHO_EPSCOR_TERRACLIMATE , population density from https://hub.worldpop.org/doi/10.5258/SOTON/WP00535 , rice prices from World Bank https://www.worldbank.org/en/research/commodity-markets). Several datasets have been data published previously and were provided by the authors (Community Forest Managed Area boundaries and covariate data (distance from roads, cities, villages, cart tracks) from Rasolofoson et al. 2015, forest cover in 2000 from Vieilledent et al. 2018).
Timing and spatial scale	Secondary spatial datasets span the period 2005-2020. Spatial scale of the analysis is Madagascar (national-scale), and all analyses were performed at two spatial resolutions (90 meters and 270 meters).
Data exclusions	Sample points from sites where CFM and MNP boundaries overlapped were excluded from the analysis, since such sites have overlapping designations. This criterion was pre-established.
Reproducibility	Data and results are available: https://zenodo.org/record/8132923. Code used for this analysis can be found at: https://github.com/raenb0/madagascar.
Randomization	Sample and comparison points were generated using the "create spatially balanced points" tool in ArcMap which generates a random sample of points that roughly represent the same proportion of the total study area.

Blinding | NA

Did the study involve field work? ☐ Yes ☒ No

Reporting for specific materials, systems and methods

We require information from authors about some types of materials, experimental systems and methods used in many studies. Here, indicate whether each material, system or method listed is relevant to your study. If you are not sure if a list item applies to your research, read the appropriate section before selecting a response.

Materials & experimental systems

n/a	Involved in the study
☒	☐ Antibodies
☒	☐ Eukaryotic cell lines
☒	☐ Palaeontology and archaeology
☒	☐ Animals and other organisms
☒	☐ Clinical data
☒	☐ Dual use research of concern
☒	☐ Plants

Methods

n/a	Involved in the study
☒	☐ ChIP-seq
☒	☐ Flow cytometry
☒	☐ MRI-based neuroimaging
